# Recent applications of chemometrics in one‐ and two‐dimensional chromatography

**DOI:** 10.1002/jssc.202000011

**Published:** 2020-03-19

**Authors:** Tijmen S. Bos, Wouter C. Knol, Stef R.A. Molenaar, Leon E. Niezen, Peter J. Schoenmakers, Govert W. Somsen, Bob W.J. Pirok

**Affiliations:** ^1^ Division of Bioanalytical Chemistry Amsterdam Institute for Molecules, Medicines and Systems Vrije Universiteit Amsterdam Amsterdam The Netherlands; ^2^ Analytical Chemistry Group van ’t Hoff Institute for Molecular Sciences, Faculty of Science University of Amsterdam Amsterdam The Netherlands; ^3^ Centre for Analytical Sciences Amsterdam (CASA) Amsterdam The Netherlands

**Keywords:** 1D chromatography, 2D chromatography, chemometrics, data processing, optimization

## Abstract

The proliferation of increasingly more sophisticated analytical separation systems, often incorporating increasingly more powerful detection techniques, such as high‐resolution mass spectrometry, causes an urgent need for highly efficient data‐analysis and optimization strategies. This is especially true for comprehensive two‐dimensional chromatography applied to the separation of very complex samples. In this contribution, the requirement for chemometric tools is explained and the latest developments in approaches for (pre‐)processing and analyzing data arising from one‐ and two‐dimensional chromatography systems are reviewed. The final part of this review focuses on the application of chemometrics for method development and optimization.

**Article Related Abbreviations**
ACO: ant‐colony optimization
ACPD: automatic chromatographic peak detection
AIC: Akaike information criterion
airPLS: adaptive iteratively reweighted penalized least squares
ALS: alternating least squares
ANN: artificial neural network
ANOVA: analysis of variance
arPLS: asymmetrically reweighted penalized least squares
asLS: asymmetrical least squares
ATLD: alternating trilinear decomposition
ATSA: automatic time‐shift alignment
BDC: Background drift correction
BEADS: baseline estimation and denoising using sparsity
BMLR: “best”‐multi‐linear‐regression
CC: corner cutting
COF: chromatographic objective function
COSHIFT: correlation optimized shifting
COW: correlation‐optimized warping
CRF: chromatographic response function
CWT: continuous wavelet transform
DAD: diode‐array detector
DTW: dynamic time warping
ECG: electrocardiography
ENALS: elastic net algorithm
FID: flame ionization detector
GC × GC: comprehensive 2D GC
HIC: hydrophobic interaction chromatography
HR: high‐resolution
iPF: iterative polynomial fitting
KSFA: key‐set factor analysis
LC × LC: comprehensive 2D LC
LC‐HRMS: LC– high‐resolution‐MS
LDA: linear discriminant analysis
LFER: linear‐free‐energy‐relationship
LMV: local minimum value
LSS: linear solvent strength
LWR: locally weighted regression
MairPLS: modified adaptive iteratively reweighted penalized least squares
MCR: multivariate curve resolution
MM: mixture model
MPLS: morphologically weighted penalized least squares
MTBSTFA: *N*‐methyl‐*N*‐(*tert*‐butyldimethylsilyl)trifluoroacetamide
MWMV: moving‐window‐minimum‐value
NBG: normal‐gamma‐Bernoulli
NEB: normal‐exponential‐Bernoulli
NLLSCF: non‐linear least squares curve fitting
NPLC: normal‐phase LC
OPA: orthogonal projection approach
OPLS‐DA: orthogonal‐partial‐least‐squares‐discriminant analysis
OSP: orthogonal subspace projection
OSSP: orthogonal spectral signal projection
PARAFAC: parallel factor analysis
PCA: principal component analysis
PCB: polychlorinated‐biphenyl
PCC: Pearson correlation coefficient
PDR: projected‐difference‐resolution
PEWS: predictive elution‐window shifting and stretching
PIOTR: program for interpretive optimization of 2D resolution
PLS‐1: single response partial‐least‐squares
PLS‐DA: partial least squares‐discriminant analysis
POPLC: phase‐optimization liquid chromatography
PRISMA: mobile phase optimization model
PW: peak‐weighted
PWT: parametric time warping
QSSR: quantitative‐structure‐retention‐relationship
REP: resolution‐enhanced peak
RF: random‐forest
RID: refractive‐index detector
ROI: region‐of‐interest
RSA: robust statistical analysis
RWPD: recursive wavelet peak detection
SIMCA: soft independent modeling of class analogy
SIMPLISMA: simple‐to‐use self‐modeling analysis
sLC × LC: selective comprehensive 2D‐LC
SSQ: sum of squares
SVD: singular value decomposition
SVM: Support vector machines
TAG: triacylglycerol
TIC: total‐ion‐current
ToF: time‐of‐flight
TPC: total peak correlation
Tri‐PLS: trilinear partial least squares
XRD: X‐ray diffraction

## 1 INTRODUCTION

Analytical instruments are indispensable for modern society. To keep pace with the growing needs of society to obtain extended and reliable information on an increasing number of sample characteristics, analytical methods are continuously improved [1]. New analytical tools typically are able to generate more and more complex data, from which it is increasingly difficult to extract useful information and deduce simple and correct answers, especially when multi‐component samples are analyzed. To extract all valuable information from what has been referred to by some as “a tsunami of data” or, more generally, “Big Data,” efficient data‐analysis strategies are evidently needed [2].

One frequently applied analytical tool is chromatography, where the separation of analytes in a mixture may be obtained by exploiting differences in their partitioning between the employed stationary and mobile phases. The employed detection techniques can detect one signal as a function of time, often referred to as single‐channel data, or a spectrum at every point in time. This multi‐channel data may facilitate identification or quantification of the analyte represented by the chromatographic signal. Although co‐elution of multiple analytes upon chromatographic analysis may significantly complicate quantification and identification [3, 4].

**FIGURE 1:**
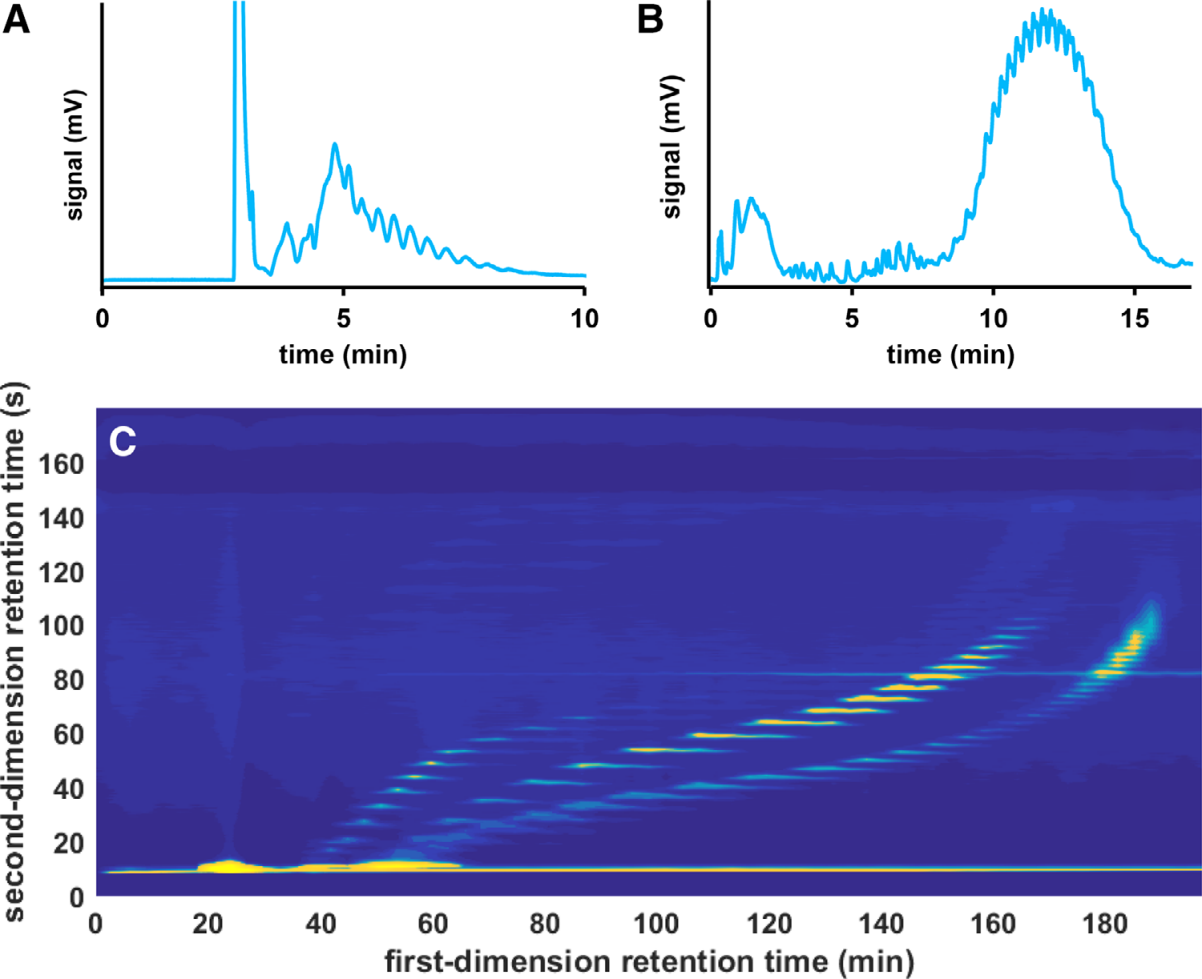
Separation of a mixture of industrial surfactants using (A) mixed‐mode ion‐exchange LC, (B) reversed‐phase LC, and (C) a comprehensive combination of mixed‐mode ion‐exchange LC and reversed‐phase LC. Adapted with permission from [7]

The quest for more separation power led to the development of comprehensive 2D chromatography where the entire first‐dimension (^1^D) effluent is divided into many fractions, each of which is subjected to a second‐dimension (^2^D) separation [5, 6]. The result is illustrated for a comprehensive 2D LC (LC×LC) separation in Figure 1, where a mixed‐mode ion‐exchange LC separation (A) is combined with a reversed‐phase LC separation (B) leading to a 2D chromatogram (C) [7]. Qualitative information may be obtained from the position of the spots (potentially supported by data obtained from MS detection) and quantitative information from the spot intensities [8].

However, when applied to highly complex samples even with 2D chromatography, it can still be difficult to extract accurate and correct information from the obtained results. Indeed, samples such as copolymer formulations [9, 10], food [11, 12], protein digests [13, 14], metabolic mixtures [15], and oil mixtures [16, 17, 18] may easily contain thousands of different components. To resolve these, powerful separation systems are needed, often equipped with sophisticated detectors such as high‐resolution mass spectrometers that are able to generate huge amounts of higher‐order data [19]. A large amount of information is contained in the resulting datasets, with a mass spectrum (and sometimes multiple fragmentation spectra) at each point in time in the 2D separation space. Arguably, extracting all relevant information is the biggest challenge we currently face in high‐resolution chromatography. Fortunately, many researchers are devoting their time to developing efficient chemometric data‐processing strategies.

In this article, we review the latest developments in the field of chemometrics applied to 1D and 2D chromatography. First, preprocessing methods will be discussed in which we address post‐analysis corrections to resolve baseline drift, undesired background signals, shifting retention times, and unresolved peaks. The second part of the review will focus on the interpretation of the data, commonly referred to as data analysis, or information extraction. In some cases, proper interpretation of the data can lead to new insights that may be used to further improve the analytical method. The final part of the review will, therefore, focus on the application of chemometrics for method development and optimization.

It is worth mentioning that, ultimately, 2D chromatographic datasets comprise a collection of 1D separations. Consequently, many of the chemometric strategies used in 2D chromatography are based on the analysis of 1D chromatograms.

## 2 PREPROCESSING

### 2.1 Aim

The main data preprocessing strategies are generally considered to be (i) denoising and smoothing, (ii) baseline (drift) correction, (iii) retention time alignment, (iv) peak deconvolution and resolution enhancement, and (v) data compression. Steps (i) and (ii) together are generally termed “background correction” and are required for the accurate identification and, especially, quantification of analytes. This has been a long‐standing issue, with the first reports having been published in the 1960s [20, 21]. During the denoising and smoothing procedures, low‐amplitude signals are first removed, irrespective of their frequency spectrum, after which high‐frequency signals are removed, irrespective of their amplitude. Next, baseline (drift) correction can be performed, with the aim to determine the baseline shape and subtract it from the measurement. Step (iii), retention time alignment, is used to correct shifts in retention time that occur between experiments. This is required to compare a series of chromatograms and to allow one to discern the real differences between similar samples. Peak deconvolution and resolution enhancement (iv) are utilized to resolve two or more (partially) overlapping signals. Finally, data compression (v) is generally required for large datasets to both reduce the computational resources required and to speed up data analysis.

Important to note here is that all preprocessing strategies tend to rely on assumptions or premises, which, in some cases, may lead to incorrect conclusions. A case in point is background correction, which may lead to the removal of true signals. This is likely to occur when real peaks cannot easily be distinguished from the background signal. Another example is inaccurate alignment, which may occur due to the incorrect identification of landmark peaks (or anchor points) used for the alignment. This can subsequently lead to errors during data analysis when assessing the differences between chromatograms. It should also be stressed that, while a preprocessing method may yield correct results in a specific situation, its usefulness should always be critically assessed for any other application, lest incorrect conclusions are drawn. This section reviews recent developments regarding the preprocessing of chromatographic data, with a focus on recent strategies for background correction and retention‐time alignment. Where useful, less‐recent methods are also briefly explained.

### 2.2 Baseline correction

As described previously, the first preprocessing step involves denoising, smoothing, and baseline‐drift correction to reduce baseline disturbances. In LC, noise mainly results from small fluctuations in the flow rate, the mobile‐phase composition, and the temperature. Drift results primarily from a variation in the mobile‐phase composition (gradients). In GC electronic noise may dominate and drift arises from the variations in the flow rate and temperature‐induced “bleeding” of the stationary phase. Certain derivative‐based peak detection methods (see Section 3.1) may struggle when such noise is present, illustrating the necessity for noise removal. In this paper, well‐known noise removal strategies, such as Savitsky–Golay [22] or Kalman filtering [23], are not specifically discussed. However, many of the recent background correction procedures either perform such noise removal prior to base‐line drift correction or utilize subsequent peak detection methods that do not require noise removal. The baseline‐drift correction is often performed by either a curve‐fitting or a smoothing strategy [24]. The aim in both approaches is to fit a curve through the presumed background data points, by utilizing a loss function, such as the well‐known least‐squares, or by polynomial fitting [24]. Background correction methods can be roughly categorized as parametric or nonparametric. Parametric models are defined as those models that assume the background is of a certain form that can be described by a constant number of parameters, e.g. linear, quadratic, or polynomial regression. Nonparametric methods, on the other hand, make no prior assumptions regarding the shape of the baseline and allow for a flexible number of parameters, the exact number of which depends on the data. Many background correction methods are nonparametric, these include adaptive iteratively reweighted penalized least squares (airPLS), asymmetrical least squares (asLS), and corner cutting (CC) [25]. Interpolation may in some cases also be required when the actual shape of the background signal under the peaks must be determined. When a large number of peak clusters are present, baseline correction can become increasingly difficult, as the data points that contain information on the background become scarce. However, as stated above, such baseline‐less data sets are becoming increasingly common with the ever‐increasing complexity of the samples analytical chemists are asked to deal with. Certain techniques perform especially well in these cases (see Section 2.2.5) [26].

#### 2.2.1 Penalized least squares approach

Many background correction algorithms are based around the use of penalized least squares, which is a smoothing method based on the Whittaker smoothing function [27]. Such methods include adaptive iteratively reweighted penalized least squares (airPLS), modified airPLS (MairPLS), asymmetrical least squares (asLS), asymmetrically reweighted penalized least squares (arPLS), and morphologically weighted penalized least squares (MPLS) [24, 28, 29].

The penalized least squares algorithm relies on balancing the fit of a model to the data, *F*, given by the sum of squares (SSQ), against its roughness (*R*) by adjusting a smoothing parameter, λ. This is given by:

(1) $$\begin{array}{ccc} Q & = & F+\lambda R=\sum_{i=1}^{m}{\left(x_{i}-z_{i}\right)}^{2}+\lambda \sum_{i=2}^{m}{\left(\Delta z_{i}\right)}^{2}\\ & = & {\left\|x-z\right\|}^{2}\;+\lambda {\left\|\mathrm{Dz}\right\|}^{2} \end{array}$$

where $x_{i}$ is the *i*th data point in the signal (x), D is the derivative of the identity matrix (I), and $z_{i}$ is the *i*th point of the fitted data, z. Solving for $\frac{\partial Q}{\partial z}=\;0$ returns a set of linear equations that can be solved to determine the fit, z:

(2) $$\left(I+\lambda D^{\prime }D\right)z\;=\;x$$

To utilize this smoothing function for baseline correction, one must first establish the location of peaks in the chromatogram. Once these peak points are known, a binary mask or “weighted matrix” can be created, the points of which correspond to either one or zero, depending on whether the data point in the chromatogram corresponds to background or to a peak, respectively. This is the approach taken by both Cobas [30] and Zhang et al. [31].

(3) $$\left(W+\lambda D^{\prime }D\right)z\;=\;\mathrm{Wx}$$

with W the weighted matrix or binary mask indicating the location of peaks. The disadvantage of this weighted‐least‐squares method is that it requires peak detection, which may in itself be affected by the correct definition of the baseline. The asymmetrical least squares (asLS) method developed by Eilers et al. [24] aims to solve this issue by introducing an asymmetry parameter. This parameter allows for the weights that are placed on positive and negative deviations from the baseline to be smaller and larger, respectively. However, in the case of asLS, this asymmetry parameter is constant, irrespective of the position on the baseline. For this reason, airPLS was introduced [29], which allows for certain regions of the baseline to be penalized more than other regions. In airPLS, a weight vector is obtained by iteratively solving a weighted penalized least squares problem. An accurate weight vector is thought to be established once the difference between the signal and the fitted vector $|d_{t}|$ falls below one thousandth of the original signal.

(4) $$\left|d_{t}\right|<0.001\left|x\right|$$

Both asLS and airPLS overestimate the baseline in the presence of additive noise. Therefore, the asymmetrically reweighted penalizes least squares (arPLS) approach was developed by Baek et al. [32]. Additional methods based around the same principles are MairPLS, in which the chromatogram (x) is pretreated prior to performing airPLS (see Section 2.2.4) [29], and MPLS, developed by Li et al. For MPLS a morphological strategy is used for the initial determination of the weight vector [28, 33]. Background drift is ultimately accounted for by using the previously described weighted penalized least squares.

While the penalized least squares approaches are not considered computationally intensive, it should be noted that all of them require finding the correct smoothing (λ) parameter to fit the baseline. This may make these methods more time consuming than some of the other methods.

#### 2.2.2 Multivariate curve resolution and orthogonal subspace projection for background correction

Multivariate curve resolution (MCR‐ALS) is one of the best‐known two‐way data analysis methods. It allows recovering the number of components in a mixture, their response profiles, and their estimated concentrations [34, 35]. Therefore, MCR is often applied for quantitative purposes (see Section 3.4). However, it may also be used for background correction. MCR requires the data to satisfy the condition of bilinearity. Examples of its application include LC–DAD and LC–MS data [36, 37]. MCR decomposes a matrix into pure chromatographic and spectral profiles, plus noise or error, as in equation (5)

(5) $$X\;=CS^{T}\;+E$$

in which *X* represents the recorded data, and *C* and *S* the pure chromatographic and spectral profiles of the components in the sample, respectively. *E* is the error matrix, (ideally) containing only instrumental noise. Often initial estimates are made by singular value decomposition (SVD) [38] or PCA, but sometimes alternative methods are used [39]. Then constraints are set in place and the equation is iteratively optimized by means of alternating least‐squares (ALS). The signal *X* does not only contain information on analytes but also on background drift:

(6a) $$X\;=X_{\mathrm{analyte}}\;+X_{\mathrm{background}}$$

(6b) $$X_{\mathrm{analyte}}=\;c_{1}{s_{1}}^{T}+c_{2}{s_{2}}^{T}\cdot \cdot \cdot c_{N}{s_{N}}^{T}$$

(6c) $$X_{\mathrm{background}}=c_{bk,1}{s_{bk,1}}^{T}+c_{bk,2}{s_{bk,2}}^{T}\cdot \cdot \cdot c_{bk,M}{s_{bk,M}}^{T}\;$$

By considering that the spectra of the analytes $s_{N}$ also contain background data, a subspace projection can be created that is orthogonal to the original data. Multiplication of the original data with this subspace will cause the background drift to be canceled out, which is called orthogonal subspace projection (OSP) or orthogonal spectral signal projection (OSSP). For more information regarding this technique and its use in background correction, please refer to the literature [40, 41].

#### 2.2.3 Corner cutting with Bezier smoothing

One example of nonparametric background correction is the CC method that has been developed by Liu et al. [25]. In CC, a smooth baseline is generated by fitting a Bezier curve [42] through the points that remain after corner points are removed from the signal vector. These corner points are defined as those points that lie above a straight line created between the previous and subsequent points in the data. This results in the automatic removal of peaks as these, by definition, will be corner points. However, a disadvantage of the approach is that it results in increasingly concave baselines as the algorithm progresses. This has been addressed by the authors by introducing a terminal condition related to the average area reduction that occurs during the iterations. The baseline is obtained after the iteration at which the average reduction in area is maximal. The approach was evaluated by comparing it to airPLS and various software packages [43, 44, 45], as well as by employing support vector machines (SVM; see Section 3.3.4) classification. Since improved baseline correction should lead to better classification results, this may be one criterion to decide which method performs best. By correcting the background in Raman, X‐ray diffraction (XRD), LC–MS, and matrix‐assisted laser desorption/ionization–time‐of‐flight MS (MALDI‐ToF MS) data, the CC method was shown to yield the best results, without requiring additional parameters to be determined.

#### 2.2.4 Local minimum value approach

Another approach to baseline correction is by utilizing the concept of local minimum values (LMVs) [46]. The approach consists of three stages, namely: (i) initialization, (ii) iterative optimization, and (iii) an estimation of background drift. In the first stage, a set of data points are assigned as local minimum values if the following set of conditions are satisfied:

(7a) $$p_{i-1}>p_{i}$$

(7b) $$p_{i}<p_{i+1}$$

**FIGURE 2:**
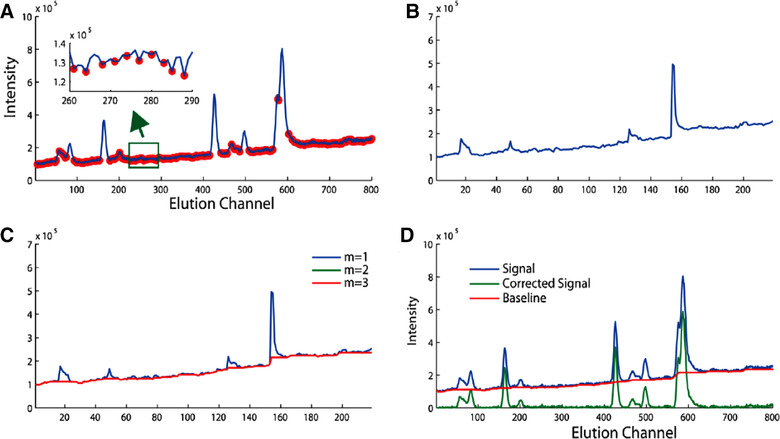
Background correction using LMVs, (A) The selection of LMV's by the criteria of Eqns. 7a and 7b, (B) The resulting minimum vector, (C) removal of outliers by a moving‐window strategy, with m the respective iteration, and (D) the original signal, the baseline, and the signal corrected for background. Reproduced with permission from [46]

In which $p_{i}$ is the *i*th data point in the chromatogram, while $p_{i-1}$ and $p_{i+1}$ are the data points before and after $p_{i}$. A chromatogram with LMVs selected is illustrated in Figure 2.

The complete set of LMVs is stored in a “minimum vector” and consists of chromatographic peak points and noise. This minimum vector is shown in Figure 2B. Any of the peak points that may have been included in the minimum vector are treated as outliers and removed by utilizing a moving‐window strategy. This requires an a priori estimation of the appropriate width of the moving window. The initial minimum vector that still contains outliers and the corrected minimum vector are shown in Figure 2B and C, respectively. Any point with an S/N ratio larger than 2.5 is considered a peak point and replaced with the median value of an extracted vector from the window in which that point occurs. This strategy is then repeated until convergence. After the iterative optimization stage, the baseline is estimated by linear interpolation. The corrected chromatogram is ultimately obtained by subtracting the estimated baseline from the original data, as is illustrated in Figure 2D.

The LMV method was compared with morphological‐penalized‐least‐squares (MPLS) [28] and moving‐window‐minimum‐value (MWMV) methods [47] using both simulated and GC data. The simulated data consisted of both single and overlapping peaks, with the latter being composed of contributions of two, three, or four peaks. Using the simulated data, peak areas and SDs were determined after background correction by local minimum values‐robust statistical analysis (LMV‐RSA), MWMV and MPLS at different levels of noise. It was demonstrated that the LMV‐RSA approach yielded the most‐accurate peak areas and the lowest SDs, with recoveries close to 100% in all cases and SDs below 4.5% at all but the highest noise level. MWMV performed slightly worse, while MPLS generally resulted in significantly lower peak areas, especially in the case of overlapping peaks, with recoveries of around 53 and 74% for the peak clusters containing three and four peaks, respectively. The influence of the moving‐window width (in the range between 20 and 80 data points) was found negligible for the GC data set.

Additionally, the LMV approach was compared to the “background drift correction by orthogonal subspace projection” (BD‐OSP) method, which was utilized for the LC‐QTOF‐MS data [41]. In this case, the differences were only assessed qualitatively. It was shown that after correction with BD‐OSP, total‐ion‐current (TIC) data still contained background drift, whereas data corrected with LMV‐RSA did not contain background drift but had lost part of the information contained in the TIC [44]. The comparisons showed that LMV‐RSA performed comparably or better than the MPLS, MWMV, and OSP approaches. However, as also stated by the authors, it is important to note that the technique can only be applied if local minimum values can be assigned.

#### 2.2.5 Automatic peak detection and background drift correction

Another approach to automated background correction combined with peak detection is the automatic peak detection and background drift correction (ACPD‐BDC) method of Yu et al. [48] First, peak start points ($x_{i})$ and endpoints ($x_{j})$ were determined. A data point was defined as a start point if the following condition was satisfied:

(8) $$x_{i}<x_{i+1}<x_{i+2}<x_{i+3}$$

i.e. starting position of a peak $x_{i}$ must be smaller than the next three data points, $x_{i+1}$ to $x_{i+3}$. Similarly, any data point is defined as an endpoint of the peak if the following condition is satisfied:

(9) $$x_{j}>x_{j+1}>x_{j+2}>x_{j+3}$$

which similarly states that a peak's endpoint $x_{j}$ must be larger than the next three points, $x_{j+1}$ to $x_{j+3}$. While not stated explicitly by the authors it is assumed by us that in condition (8) only the first point in an increasing series is taken as a peak starting point, as this condition will lead to multiple points of increasing intensity being detected while the signal is rising, depending on peak width and detector frequency. Similarly, for condition (9), only the last point in a decreasing series should be taken as a peak's endpoint. These start and end points were then contained in two vectors (a = [*a*_1_ *a*_2_ … $a_{p}$] and b = [*b*_1_ *b*_2_ … $b_{q}$]). A combination of a starting and ending point, [$a_{m}\;b_{n}$], was considered a peak's elution range as long as the following logical condition is met: $b_{n-1}<a_{m}<b_{n}<a_{m+1}$. All detected peaks were subsequently subtracted from the original signal, ***x***. In this way, an initial estimate of the background was made ($x_{new}$). Threshold values were established using the first‐order derivative of this initial estimate ($dx_{new}$) and outliers were iteratively removed by condition (Eq. 10), with noise thresholds being defined as 3σ.

(10) $$\frac{\left|d_{i}-\bar{dx_{new}}\right|}{\sigma }>3$$

**FIGURE 3:**
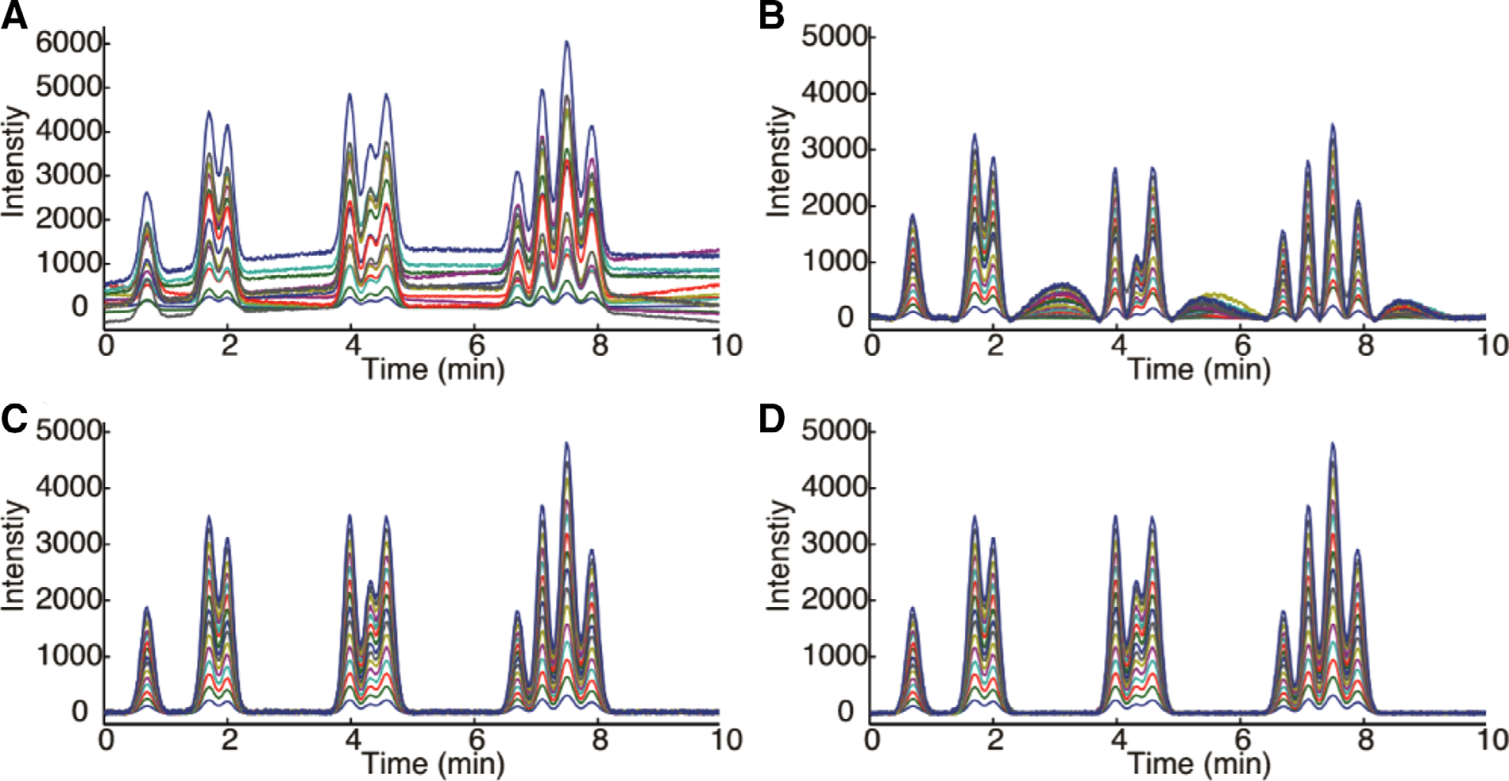
Comparison of background drift correction in 15 LC samples, containing 11 antibiotics in tap water. (A) Original chromatograms, (B) and (C) background correction by airPLS and MairPLS, respectively (smoothing factor, $\lambda \;=10^{4}$), (D) correction by ACPD‐BDC. Reproduced with permission from [48]

In which σ is the SD within $dx_{new}$ and $d_{i}$ is the *i*th element of $dx_{new}$. This condition estimates the noise level, by iteratively removing elements in $dx_{new}$. It is important to obtain a correct $dx_{new}$ vector, as its first‐order derivative is subsequently used as a threshold to selectively remove pseudo peaks from the original signal (***x***). This was carried out by evaluating the first‐order and second‐order derivatives of the original signal. Pseudo peaks were removed based on two conditions, i.e. (i) the absolute value of the first‐order derivative of the original signal, relative to the threshold value previously established using Eq. 10, and (ii) the number of times the second‐order derivative crosses the zero‐line. The authors accepted a signal as a true peak if the absolute value of the first‐order derivative was five times larger than the noise threshold, and if the second‐order derivative crossed the zero‐line fewer than eight times. Background drift was ultimately corrected for by first replacing the previously detected regions containing peaks by linear baselines and was denoised using three‐point moving‐window averaging. This resulted in a modified signal vector ($x_{background}$), which is now assumed to accurately describe the background. Baseline correction is then performed by subtracting this background from the original signal. The developed background correction procedure was then evaluated and compared to the use of airPLS [29] and MairPLS, in which the background signal ($x_{background}$) is used rather than the original chromatogram signal (***x***) as in airPLS. These three methods were applied for the background correction of simulated data, experimental LC data on a sample containing 11 antibiotics in tap water, and GC data on plant‐based flavor extracts. MairPLS and ACPD‐BDC performed similarly for all data sets evaluated, while airPLS performed considerably worse. This is illustrated in Figure 3 where for each method the uncorrected and background‐corrected LC chromatograms are shown.

MairPLS and ACPD‐BDC were further evaluated by means of PCA (see Section 3.2.1.1). In this study, the variance explained by the first principal component before and after background correction is used as figures of merit. For the LC data, this increased from 36.9% before background correction to 43.5% after background correction by MairPLS and to 44.4% when ACPD‐BDC was used. For the GC data set, almost no change was observed in the percentage of variance explained, which remained close to 95.0% in all cases.

#### 2.2.6 Bayesian approaches to background correction

**FIGURE 4:**
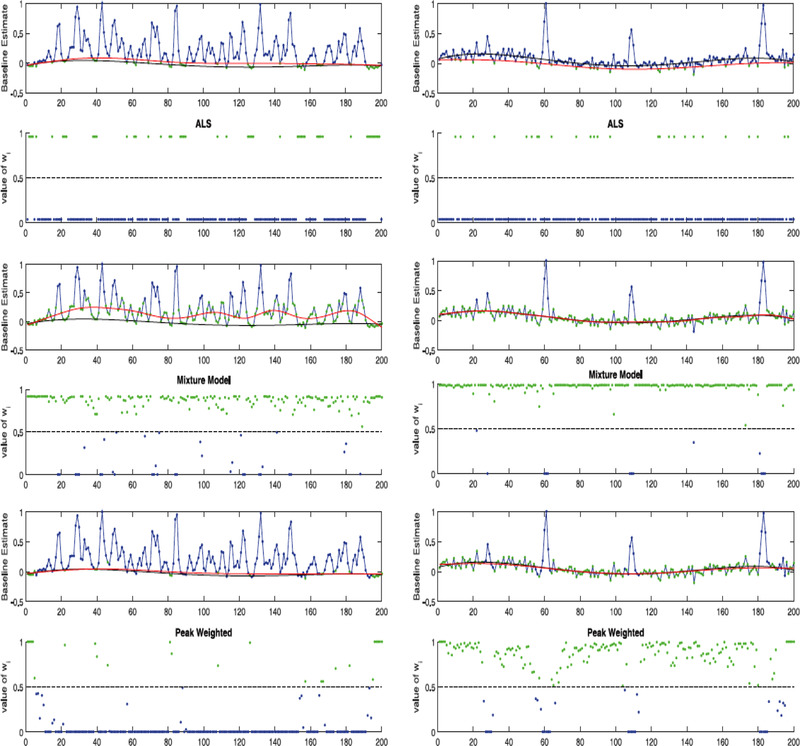
Comparison of background‐drift correction by asLS, MM, and PW methods for crowded (left) and sparse (right) simulated chromatograms [26]. The green points are those points that have been given high weight by the PW model and are primarily used to describe the background, while the blue points have been given low weights

As previously stated, baseline correction is often hindered by crowded chromatograms and low S/N ratios. One approach aimed specifically at facilitating baseline correction even under these conditions has been developed by Lopatka et al. [26]. In this approach, a probabilistic peak‐detection algorithm is used to determine the probability of a point in the chromatogram belonging to a peak or to the baseline. It is hence termed the peak‐weighted (PW) method. The algorithm operates by fitting several different models across a set window of data using a least‐squares approach. Then, a likelihood is assigned to each model and from this, the probability of the data point belonging to a peak is calculated. User‐defined parameters include the number of overlapping peaks allowed in each section and the window width, which directly depends on the peak width. This approach was compared to the mixture model (MM) and asymmetrical least‐squares (asLS) [24, 49] approaches and was shown to perform especially well in the case of crowded chromatograms. This is illustrated for simulated data in Figure 4.

The PW method was also applied for background correction of a comprehensive 2D GC‐FID chromatogram of fire debris. However, with suitable benchmarks unavailable, the authors found it impossible to objectively assess the performance of the PW method in this situation.

A different approach based on Bayesian regularized artificial neural networks (BRANN) [50] was developed by Mani‐Varnosfaderani et al. The iterative BRANN algorithm was compared to airPLS, MPLS, iPF, and CC (see Sections 2.3.2) methods using the projected‐difference‐resolution (PDR) criterion.

#### 2.2.7 Baseline estimation and denoising using sparsity

When a signal can be described reasonably accurately using only a few non‐zero parameters it can be classified as sparse. For a typical chromatogram, consisting of peaks, noise, and background, this assumption may also be applied if it features relatively few peaks compared to the number of baseline points. One algorithm that utilizes this concept of sparsity, and has been developed recently, is called baseline estimation and denoising using sparsity (BEADS) [51]. It was later further improved to create the “assisted BEADS” algorithm [52]. BEADS specifically aims to model the signal, background, and noise, without employing the use of overly restrictive parametric models. As the background is considered a low‐pass signal, depending on the cut‐off frequency, low‐pass filters may allow this background to be removed. In mathematical terms, the approach is based on modeling the chromatographic signal as:

(11) y=s+w=x+f+w

with *y* the input data or chromatogram containing peaks *x*, baseline *f*, and white Gaussian noise *w*. Thus *s* describes the noiseless input chromatogram (x+f). It is assumed that in the absence of peaks, the baseline can be estimated by utilizing a low‐pass filter. Thus, from an estimate of the peak vector ($\hat{x})$ an estimate of the baseline ($\hat{f})$ can be obtained by filtering the chromatogram.

(12) $$\hat{f}=\;L\left(y-\hat{x}\right)$$

Once the baseline is estimated, the noiseless input chromatogram ($\hat{s}$) can also be obtained as this is simply $\hat{x}+\hat{f}$. This means $\hat{s}$ can be estimated by using both a low‐pass filter L and a high‐pass filter H.

(13) $$\hat{s}=Ly+H\hat{x}$$

The task is then to obtain an accurate estimate of the peak vector and to establish suitable filters. To achieve this, the authors investigated two different cost functions and employed an algorithm to minimize these. For a more extensive overview of the cost functions and algorithm employed, please refer to Ning et al. [51].

**FIGURE 5:**
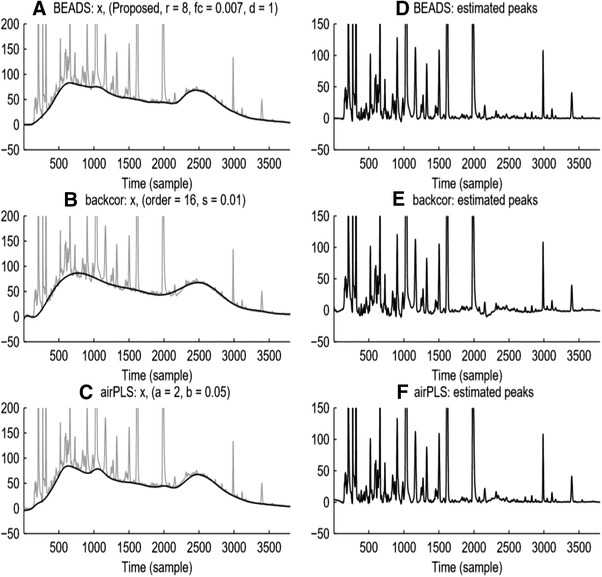
Comparison of background drift correction using BEADS (top), backcor (middle), and airPLS (bottom). Reproduced with permission from [51]

The performance of BEADS was compared to airPLS [29] and backcor [53] strategies for baseline correction of simulated and real chromatographic data. The results are illustrated in Figure 5, using chromatographic data from Zhang et al. [29].

BEADS was found to have performed favorably in comparison with airPLS and backcor, with the former underestimating the baseline in the range from datapoint (sample in Figure 5) 2200 to 2500 and the latter overestimating the baseline in this region. However, while BEADS performed well, the baseline was required to be periodic, i.e. the signal at the start of the chromatogram should be equal to that at the end of the chromatogram. If the above requirement is not fulfilled, for example, due to changes in mobile‐phase composition or temperature, the modeled baseline will show end‐point transient artifacts. This would manifest in a decrease (or increase) of the baseline toward the starting value at the endpoint of the chromatogram. An additional limitation includes the need to manually adjust parameters, such as the order of the filter employed and its cut‐off frequency, the penalty function utilized in the optimization and its asymmetry, and the regularization parameters, which should be set in accordance with the expected sparsity of the data. Small changes in these parameters can result in very different baselines. However, as noted by the authors, these parameters do allow the approach to be used for diverse signals, including, for example, baseline estimation in electrocardiography (ECG). Furthermore, BEADS cannot correctly handle negative signals, such as those observed in, for example, refractive‐index detection (RID).

To summarize, the following difficulties arise when using BEADS for baseline correction: (i) parameter adjustment and selection (ii) the signal intensity for the first and last points in the chromatogram should be equal, and (iii) difficulties with assessing data that may contain negative peaks. Most of these limitations have been addressed by Navarro‐Huerta et al. [52] who have developed the assisted‐BEADS algorithm, and by Selesnick, who has proposed a solution for the endpoint artifacts [54]. Parameter selection may be facilitated by auxiliary autocorrelation plots. In such plots, the correlation between consecutive data points is measured. By determining the autocorrelation of the noise after background correction and by plotting this as a function of one (or, ideally, all) of the adjustable parameters, the optimal value of the parameter(s) can be established from the location in the plot where autocorrelation is minimized. To address the sensitivity of BEADS to negative peaks, an additional algorithm has been applied, which discards sporadic negative signals [52].

#### 2.2.8 Background correction in GC–MS and LC–MS using recorded profile spectra

An MS‐based approach to baseline correction and noise removal in GC–MS and LC–MS data has been developed by Erny et al. [55]. In this work, the recorded profile (full) spectra were used rather than conventional centroid mass spectra. The latter is obtained by retaining only the peak centers at discrete *m*/*z* values (i.e. zero‐line width), and the corresponding intensity while discarding any other information. However, it has recently been shown that errors may result from the use of centroided spectra in subsequent data analysis, the most prominent being the merging of overlapping peaks. As the number of profile spectra to be analyzed was 60 000 and 141 000 for CE–ToF‐MS and UHPLC–QToF‐MS, respectively, a selection of profile spectra was first made based on their relative length. This relative length is defined as the number of non‐zero values divided by the total number of values in the MS profile. As a zero value means no ion is detected at the given time and *m*/*z* interval, the relative length is an indicator of what type of information is contained within the profile. By generating a base peak profile from a selection of profiles that differ in relative length, the information in these profiles can be visualized. Using this approach, the authors selected the profile spectra with a relative range of 75–100% as the data to use for background correction, along with profiles containing more than 50% of non‐zero values. This resulted in 3909 and 37 000 profiles for background correction in CE–ToF‐MS and UHPLC–QToF‐MS, respectively. The same strategy was also applied for noise estimation, using the profile spectra in the relative range from 0 to 25%. Both airPLS and arPLS were then investigated for baseline correction, while a moving‐window strategy was employed for noise removal using the noise estimated from the base‐peak profile as a threshold value. The use of a higher noise threshold was also investigated, however, this ultimately resulted in the removal of low‐intensity peaks. The background correction itself, performed with arPLS, did not result in significant alterations of the total‐ion profile. As a final step, the spectra are converted back to conventional MS‐centroid spectra. The computation time was approximately 2 and 20 min for the CE‐ToF‐MS (0.7 GB) and UHPLC‐Q/ToF‐MS (2.9 GB) data sets, respectively. The primary difference with other approaches is that baseline correction and noise removal are primarily based on the profile spectra, which are first selected based on their relative length, so as to improve the accuracy of the correction. This allowed the authors to reliably obtain base‐peak ions that were previously obscured by background ions. It also allowed for a substantial reduction in data size.

#### 2.2.9 Methods for 2D chromatography

A number of research groups have specifically investigated methods for 2D chromatography. One example exploits the trait of visualizing LC×LC and GC×GC separations as 2D image. In their work, Reichenbach et al. utilized a number of statistical and structural characteristics of the background signal in 2D chromatograms, including the white noise properties of noise in chromatographic signals to correct for the background [56]. Their algorithm has been applied to both GC × GC and LC × LC data using the GC Image and LC Image software tools [57, 58]. Other approaches have consulted the data from the 1D perspective. Zeng et al. used the linear least‐squares curve fitting approach combined with moving‐average smoothening to correct all 1D peaks within the 2D chromatograms [59]. Zhang et al. employed alternating trilinear decomposition (ATLD) to correct the analytical signal for the background drift of LC×LC–DAD data [60]. Self‐weighted alternating trilinear decomposition (SWATLD) and parallel factor analysis (PARAFAC) were also applied for this function.

### 2.3 Retention‐time‐alignment strategies

After the data have been corrected for the background signal alignment may be required. This is especially the case in LC, where retention‐time shifts between analyses are not uncommon. This alignment is generally performed either based on integrated peak tables or on pixel‐level chromatograms. In the latter case, the entire chromatogram is used for the alignment. When using integrated peak tables, peaks are aligned by assigning a unique identifier to each peak and assuming this to be consistent across all chromatograms being aligned. Therefore, such alignment strategies are often closely linked with other chemometrics methods that allow for both peak detection and tracking. The algorithms vary in complexity from simple scalar shift alignment, alignment to selected target peaks, local alignment, to globally optimized alignment, which automatically optimizes the alignment in multiple regions of the chromatogram. Some of the best‐known globally optimized alignment approaches are correlation‐optimized warping (COW), dynamic time warping (DTW), parametric time warping (PTW), and correlation‐optimized shifting (COSHIFT) [61, 62, 63]. Many of these algorithms have been applied in various fields, such as forensic profiling and metabolic fingerprinting [64, 65].

#### 2.3.1 Correlation‐optimized warping

In COW, the chromatogram is first divided into several local regions, which are iteratively stretched and compressed until the Pearson correlation coefficient (PCC) between the sample and the reference chromatogram is maximized. The PCC is calculated from Equation (14).

(14) $$\mathrm{PCC}=\frac{{\left(r-\bar{r}\right)}^{T}\left(x-\bar{x}\right)}{\sqrt{{\left(r-\bar{r}\right)}^{T}\left(r-\bar{r}\right){\left(x-\bar{x}\right)}^{T}\left(x-\bar{x}\right)}}\;$$

in which ***r*** is a vector describing the reference chromatogram, while*** x*** is the test chromatogram. The mean values of these are given by $\bar{r}$ and $\bar{x}$, respectively. Several input parameters are required, such as the segment length and the slack length. Adaptations to COW have also been developed, including the 2D‐COW algorithm by Zhang et al. [66] and an alternative method by Gros et al. [67], which has recently been applied for alignment of GC×GC–HRMS data [68].

#### 2.3.2 Automatic time‐shift alignment

An additional approach to time‐shift alignment, automatic time‐shift alignment (ATSA), was developed by Zheng et al. [69]. This method comprises three different steps, viz (i) automatic baseline correction and peak detection, (ii) preliminary alignment through adaptive segment partition, and (iii) a precise alignment. Baseline correction was performed by LMV‐RSA (see Section 2.2.1) and peak detection was carried out by a multi‐scale Gaussian smoothing‐based strategy (see Section 3.3.2) [70]. Then the chromatogram was divided into a number of short segments, the time shifts within which were expected to be similar. A preliminary alignment of the chromatograms was performed by first establishing a reference chromatogram. However, as noted by the authors, relying solely on maximizing PCC values can lead to misalignments, as the magnitude of the PCC value is influenced strongly by large peaks. Therefore, the preliminary alignment was performed by using the total peak correlation coefficient (TPC) instead, which is calculated from:

(15) $$\mathrm{TPC}=\left(\frac{\sum_{i=1}^{I}w_{i}\mathrm{PC}C_{i}}{\sum_{i=1}^{I}w_{i}}\right)\;\frac{I}{N}$$

in which $w_{i}$ is the weight of the *i*th‐matched peak, defined as the ratio between peak area and peak length, and *I* and *N* are the number of peaks in the test and reference chromatograms, respectively. Peak length describes the width of the peak, but in number of data points, rather than time units. Segments that were not correctly aligned were treated as outliers and were re‐aligned if they did not fall within the 99% confidence interval. For re‐alignment PCC values were used and the coefficient closest to the expected time‐shift value was selected as optimal. After preliminary alignment, overlapping and disconnected segments may be present in the chromatogram. These were corrected by using a warping strategy and adjusting the boundaries between segments. To ensure that the chromatogram retains the same start and endpoints after time‐alignment, a linear interpolation strategy was used. The PCC values obtained after preliminary alignment already showed significant improvement, increasing from 0.72 to 0.96.

After the preliminary alignment, the final precise alignment was carried out by first segmenting the aligned test chromatogram based on the number of chromatographic peaks present. Boundaries set in the middle between the end position of a peak and the starting position of a subsequent peak. Each segment was then aligned to the nearest reference peak based on retention time. For segments that did not contain a reference peak, the time shift was taken as the average of that of neighboring segments. Then once again warping was used to properly align the segment boundaries, as the time‐shifts caused disconnected and overlapping segments. After performing the entire retention‐time alignment procedure the correlation coefficient improved further, from about 0.96 to about 0.99.

The authors then evaluated their approach. The influence of the two pre‐estimated parameters, i.e., the initial segment size and initial time shift were investigated. Several different settings were tested, and the obtained PCC values were compared. Initial segment size was varied incrementally from 1 to 10 min and was found to result in nearly constant PCC values of approximately 0.993. However, the authors noted that larger segment sizes (> 10 min) would reduce the required computing power but resulted in drastic time‐shift changes. The initial time‐shift estimate was varied from 0.1 to 1 min and resulted in constant PCC values. The ATSA method was also evaluated by analyzing the eventual peak areas. This is especially important because a warping strategy was used, which may influence quantification. Once again, the peak areas before and after the entire alignment strategy were compared by using the obtained PCC values. The approach was shown to have a negligible effect on the determined peak area (PCC = 0.9998). However, as stated by the authors, the relative deviation increased for very small peaks. ATSA was applied in a study concerning the storage of essential oils and it was compared with COW. The experimental data suggested the degradation of the essential oils during storage. However, after alignment using either COW or ATSA, the obtained correlation coefficients suggested that no degradation had taken place. This demonstrates clearly that the use of retention‐time alignment may lead to incorrect conclusions. Thus, whether such a strategy can be applied must be critically assessed for each application.

#### 2.3.3 MS‐based peak alignment

**FIGURE 6:**
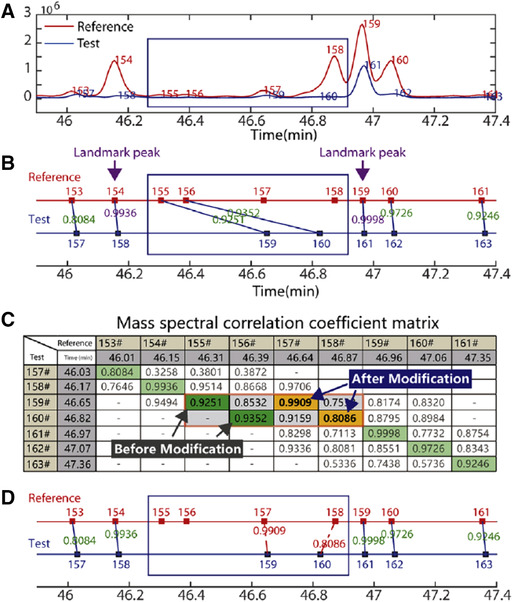
Peak alignment based on maximum‐correlation path and the additional use of landmark peaks. (A) selected range of the chromatogram; (B) misaligned peaks when only mass‐spectral information is utilized; (C) The locations of the misaligned peaks in the maximum correlation coefficient path and the modified result after utilizing landmark peaks. In green, the highest obtained PCC values are shown prior to the correction using landmark peaks; higher PCC values could be obtained by ignoring peaks 155 and 156 however, the new PCC values are shown in yellow; (D) Aligned chromatogram after correction. Reproduced with permission from [71]

Several alignment algorithms have been developed that are based on the use of MS [71, 72]. In the approach of Fu et al. [71] baseline correction was first carried out by an LMV approach (see Section 2.2.4). The actual time‐shift alignment consists of four steps: (i) extraction of the path of maximum MS‐correlation, (ii) peak‐alignment modification using landmark peaks, (iii) grouping and registration of missing peaks, and (iv) peak‐alignment refinement. The first step required an initial estimate of the time shift (0.5 min in the described case), after which PCCs (see Section 2.3.1, Eq. 14) were calculated based on mass spectra for each test and reference chromatographic peak that fell within this initial time‐shift window. All PCCs were collected in a correlation matrix that was used to determine the maximum‐correlation path. The correlation matrix and the determined maximum‐correlation path are illustrated in Figure 6.

The approach is based on the assumption that peak elution order is consistent between samples. This may not always be the case. Therefore, alignment based on landmark peaks has also been incorporated. In this approach, landmark peaks are first defined as those peaks showing PCCs above 0.99. The time shifts of these landmark peaks are then stored in a vector and outliers are removed based on the median and the standard deviations of the landmark peaks’ time shifts. Time shifts between two landmark peaks are linearly interpolated and an expected time shift can be calculated. This is then compared to the original time shift resulting from step (i) and the peak is realigned to the nearest reference peak in case the expected time shift is significantly different from the original time shift. However, as noted by the authors, while the time shift can also be approximated using non‐linear interpolation, it cannot be employed in situations where the elution order has changed. In steps (iii) and (iv), certain peaks may not be present in the reference chromatogram. These missing peaks are grouped based on their retention time with a maximum time shift window of 0.1 min, after which the chromatogram is realigned one final time.

**FIGURE 7:**
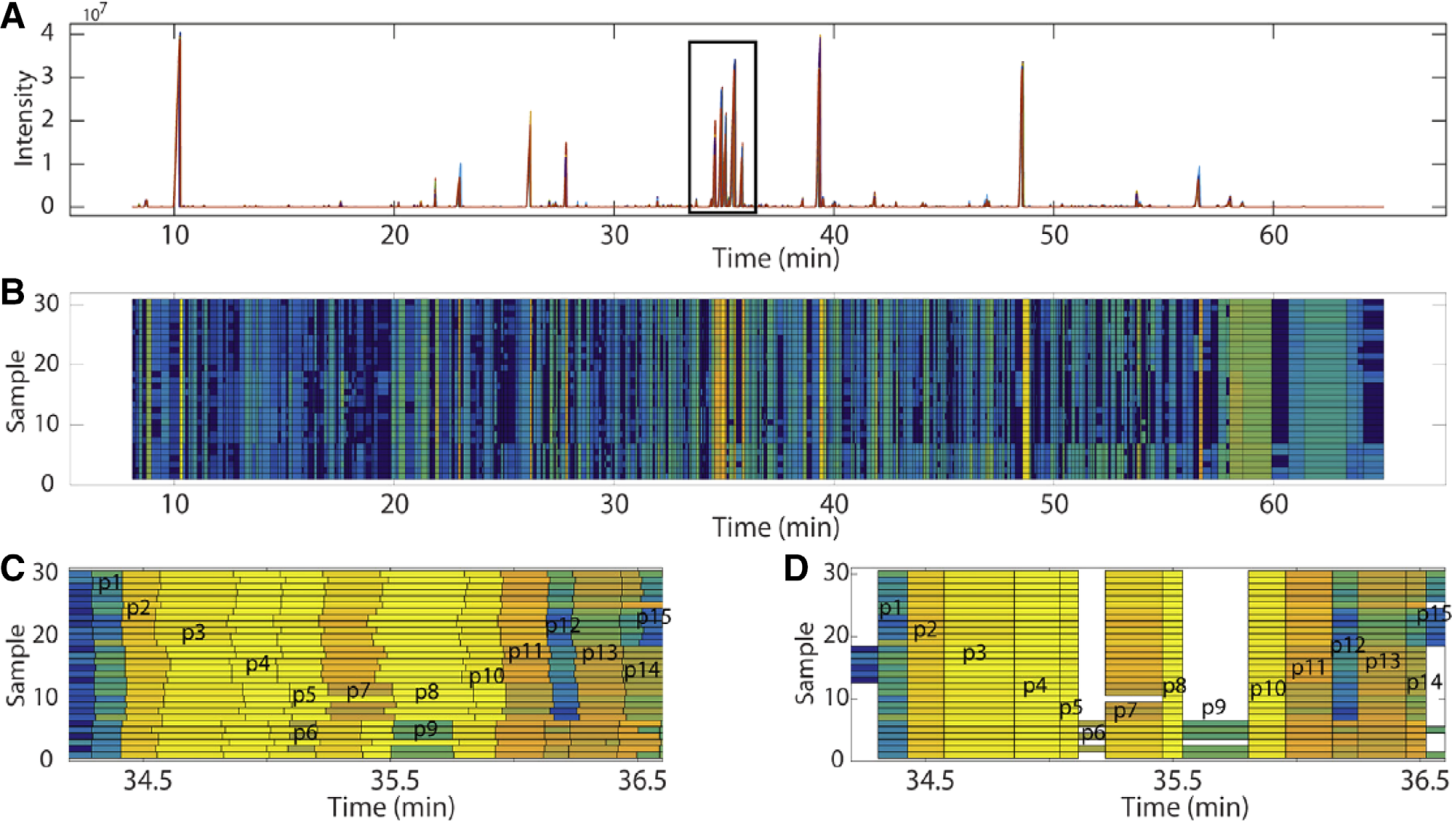
Peak alignment results with (A) the original chromatogram; highlighted are 15 closely eluting peaks, (B) alignment results, and (C) and (D) original and aligned peaks within the region containing the 15 closely eluting peaks. Reproduced with permission from [71]

The developed MS‐based alignment was validated by applying it to a GC–MS data set including 12 growth and 18 maturation plant samples. Peak‐alignment results of these 30 samples are illustrated in Figure 7 for a selection of 15 closely eluting peaks.

Another method incorporating baseline correction, peak detection, and time‐shift alignment was proposed by Yu et al. for metabolic profiling analysis of 30 plant samples [73]. The method uses ACPD for peak detection and baseline correction, after which time shifts are corrected based on the TIC data. After this pretreatment PCA, ANOVA, and PLS‐DA (Section 3.3) were applied to further analyze the data. Peak alignment required first choosing a reference chromatogram, which in this case was the chromatogram containing the highest number of peaks. After peak detection and background correction, segments from both the chromatogram to be aligned and the reference chromatogram were selected based on an initial time‐shift estimate (0.5 min was chosen). Initially, a rough alignment was performed using a similar approach as described in Section 2.3.1. In this case, the cosine correlation was calculated rather than the PCC. Note that both are related, with the difference being that the PCC is the centered cosine correlation, which itself is the normalized inner product. The sum of the weighted individual cosine values (COS) was then used to obtain the initially aligned chromatogram.

After initial alignment, a precise alignment was carried out by accounting for the relative distances, cosine values, and real distances between a chromatographic peak in the reference and each of the peaks to be aligned in the sample chromatogram, within the respective segment. This yielded an alignment table. In those cases where two of the reference peaks were aligned to the same sample peak, the peak with the smallest cosine correlation would be removed, the roles of reference and test chromatogram inverted, and the two alignment tables would be combined. For all other cases, this approach was not applied.

Although the time‐shift‐alignment procedure was validated by aligning the data from the plant samples, the procedure was not compared with other approaches. As also stated by the authors, one of the disadvantages of this peak alignment approach is that the elution order must remain unchanged between samples. This assumption is actually inherent to many of the peak‐alignment methodologies currently available.

#### 2.3.4 Approaches for 2D chromatography

In addition to the approaches above, a number of less‐recent studies have focused on retention‐time alignment in 2D chromatography where in particular second‐dimension modulations must be aligned to facilitate further data analysis. PARAFAC was applied to correct such retention‐time shifts between neighboring modulations [74]. Johnson et al. applied a windowed‐rank minimization with interpolative stretching to the separations of naphthalenes in jet fuel by GC×GC [75]. Another method applied to GC×GC data used indexing schemes for warping in both dimensions [76]. Similar to background correction, other developed methods for retention‐time alignment approached the data from an image perspective [59, 77, 78]. With most developed approaches generally exclusively adaptable to three‐way data structures, Allen and Rutan developed an approach that allowed processing of four‐way data structures and applied this to LC×LC‐DAD data [79].

##### Correction for wrap around

In some cases, analytes may not elute within the modulation time and appear in the following modulations. This is known as wrap around and is rather common in GC×GC. One method to resolve this treats the 2D chromatogram as a continuous three‐dimensional cylinder where the end of one modulation is the beginning of the next [80]. Alternatively, absolute retention times may be determined by using an integer fraction of the original modulation to detect occurrences of wrap around [81].

### 2.4 Signal deconvolution and resolution enhancement

In general, most chromatograms of complex samples suffer from overlapping signals. This problem can be reduced by utilizing efficient 2D‐LC approaches combined with selective detection techniques, such as tandem MS. However, these types of analytical systems are not always available and even when accessible, they sometimes still do not provide resolution of all components. Techniques for peak‐resolution enhancement, such as even‐derivative sharpening (see Section 2.4.1) [82], derivative symmetrization [83], or power‐law methods may be used [84, 85]. These techniques also have the potential for peak detection, as they highlight any small difference in peak shapes. However, they often struggle with noise. Other approaches, such as deconvolution or decomposition of the data, may also be applied to improve resolution beyond what is possible given the instrumental constraints. Examples include techniques such as Fourier self‐deconvolution [86], wavelets [87], and multivariate curve resolution (MCR) [3]. In many cases, these deconvolution strategies can also be applied for background correction, as they allow one to separate peaks, background, and noise vectors.

#### 2.4.1 Derivative enhancement

One recent strategy is based on even‐derivative sharpening and been developed by Wahab et al. [82] The fundamental concept is that the area of even derivatives of symmetric distributions will always be zero. It is assumed that peaks can be accurately described as either a Gaussian or a Lorentzian distribution. Asymmetric distributions are not considered and, hence, fronting and tailing are neglected. The even derivatives of a Gaussian function can be calculated analytically from

(16) $$\frac{d^{n}G\left(\sigma ,t\right)}{dt^{n}}={\left(-1\right)}^{n}\;\frac{1}{{\left(\sigma \sqrt{2}\right)}^{n}}H_{n}\left(\frac{t}{\sigma \sqrt{2}}\right)G\left(\sigma ,t\right)$$

The *n*th derivative is seen to be simply the product of the original Gaussian function *G* and a polynomial *H*. By then subtracting the 2^nd^, 6^th^ …. (2+4*m*)th order derivatives and adding the 4th, 8th … (4+4*m*)th order derivatives (where *m* is an integer), a resolution‐enhanced peak (REP) can be obtained. This is written as

(17) $$\begin{array}{ccc} \mathrm{REP} & = & G\left(1-K_{2}\frac{{\left(-1\right)}^{2}}{{\left(\sigma \sqrt{2}\right)}^{2}}H_{2}+K_{4}\frac{{\left(-1\right)}^{4}}{{\left(\sigma \sqrt{2}\right)}^{4}}H_{4}\right.\\ & & \left.-K_{6}\frac{{\left(-1\right)}^{6}}{{\left(\sigma \sqrt{2}\right)}^{6}}H_{6}+\cdots \right) \end{array}$$

The constants *K*_2_, *K*_4_, *K*_6_ … $K_{n}$ are empirically chosen to obtain sufficient resolution, while preventing significant negative dips in the baseline or a significant decrease in S/N. Generally, the 6^th^‐order and higher derivatives were not required, and the following conditions were proposed as starting points for the selection of the *K* values: $K_{2}=\frac{\sigma^{2}}{30}\;$ and $K_{4}=\frac{\sigma^{4}}{200}$.

This approach was then demonstrated on various overlapping peaks in LC analysis, namely single critical pairs, such as isomers of salbutamol and *p*‐nitro‐dl‐phenylalanine, and difficult‐to‐separate mixtures of three differently deuterated benzenes. In all cases, the resolution was improved to the extent that previously non‐baseline separated peaks became virtually baseline separated while retaining the peak area. Furthermore, the approach proved capable of detecting hidden peaks in a sample containing four steroid compounds, some of which eluted as overlapping peaks. The authors demonstrated that the approach could also be used for qualitatively improving distorted peaks. The derivative‐enhancement approach is, however, limited by the resolution between peaks ($R_{s})$. It can only be reliably used when $R_{s}>0.7$ if the final goal is the accurate quantification of overlapping peaks. If the approach is primarily used for the detection of hidden peaks this is, of course, not a requirement. For $R_{s}=\;0.74$ the error in area estimation was approximately 0.4%, whereas for lower $R_{s}$ the error quickly increased and significant baseline distortion could be observed.

#### 2.4.2 Region‐of‐interest—Multivariate curve resolution

With the advent of LC coupled to HRMS, a wealth of data can be acquired in just a single experiment. Due to the very large data sets (typically > 80 GB), filtering and compression are normally required before further data analysis can be performed. A conventional reduction approach is binning, in which the *m*/*z* axis is separated into several segments, with a width of typically a few times the mass accuracy of the mass spectrometer. Whereas such binning implies a compression of the data, it may be difficult to recover true chromatographic peak shapes from the binned data as it also implies a loss in resolution. For such cases, the region‐of‐interest (ROI) strategy has been proposed [88], which considers only certain regions with high data density. The ROIs are selected based on criteria such as signal intensity and the number of mass trace occurrences, i.e. the number of points that can be used to describe the peak in the TIC. The ROI strategy allows for compression of data, while maintaining spectral resolution. The approach is often applied in combination with deconvolution strategies, such as multivariate curve resolution–alternating least squares (MCR‐ALS, see Sections 2.2.2 and 3.4), as in this case peak alignment is not required [89]. Such an ROI‐MCR approach has been applied by Navarro‐Reig et al. [90] in combination with LC×LC–HRMS for the identification of metabolites in the rice metabolome. They selected the ROIs based on S/N ratio (0.1% of maximum MS signal intensity), mass accuracy of the MS (0.05 Da/e for the ToF mass analyzer used) and the minimum number of times the same *m*/*z* signal had to be detected consecutively (set at 25). A further compression in the time dimension was performed by means of wavelet compression [91, 92] and by a windowing strategy, separating the data into three distinct windows. Using this approach, a more than 50‐fold reduction in data size was achieved. After compression, MCR‐ALS was performed, resulting in 154 resolved metabolites, of which 139 were identified after correction.

## 3 ANALYSIS OF CHROMATOGRAPHIC DATA

After preprocessing, the focus shifts to translating complex data into useful information on a sample. Many methods for information extraction have been developed during the last decades [93, 94, 95, 96, 97, 98]. In this context, the data analysis process can be divided into several levels. First, the peaks representing the (partially) separated compounds in the sample must be detected. Comprehensive 2D chromatography requires a subsequent step of clustering the detected peaks, taking the number of modulations per first‐dimension peak into account. Next, generic information about the individual detected one‐ or two‐dimensional peaks must be extracted (e.g. area, statistical moments). Finally, the retrieved properties can be translated into useful information. In this section, we will review the latest developments for each of these steps.

### 3.1 Peak detection

The aim of peak detection is to locate true signals within the chromatogram and, therefore, it is crucial for correct interpretation of an experiment. As clarified in the introduction, a comprehensive 2D chromatogram comprises a large number of 1D chromatograms. Consequently, we will first address the detection of peaks in 1D chromatograms, because the interpretation of higher‐order chromatograms usually relies on techniques used in lower‐order data.

#### 3.1.1 Classical peak detection

**FIGURE 8:**
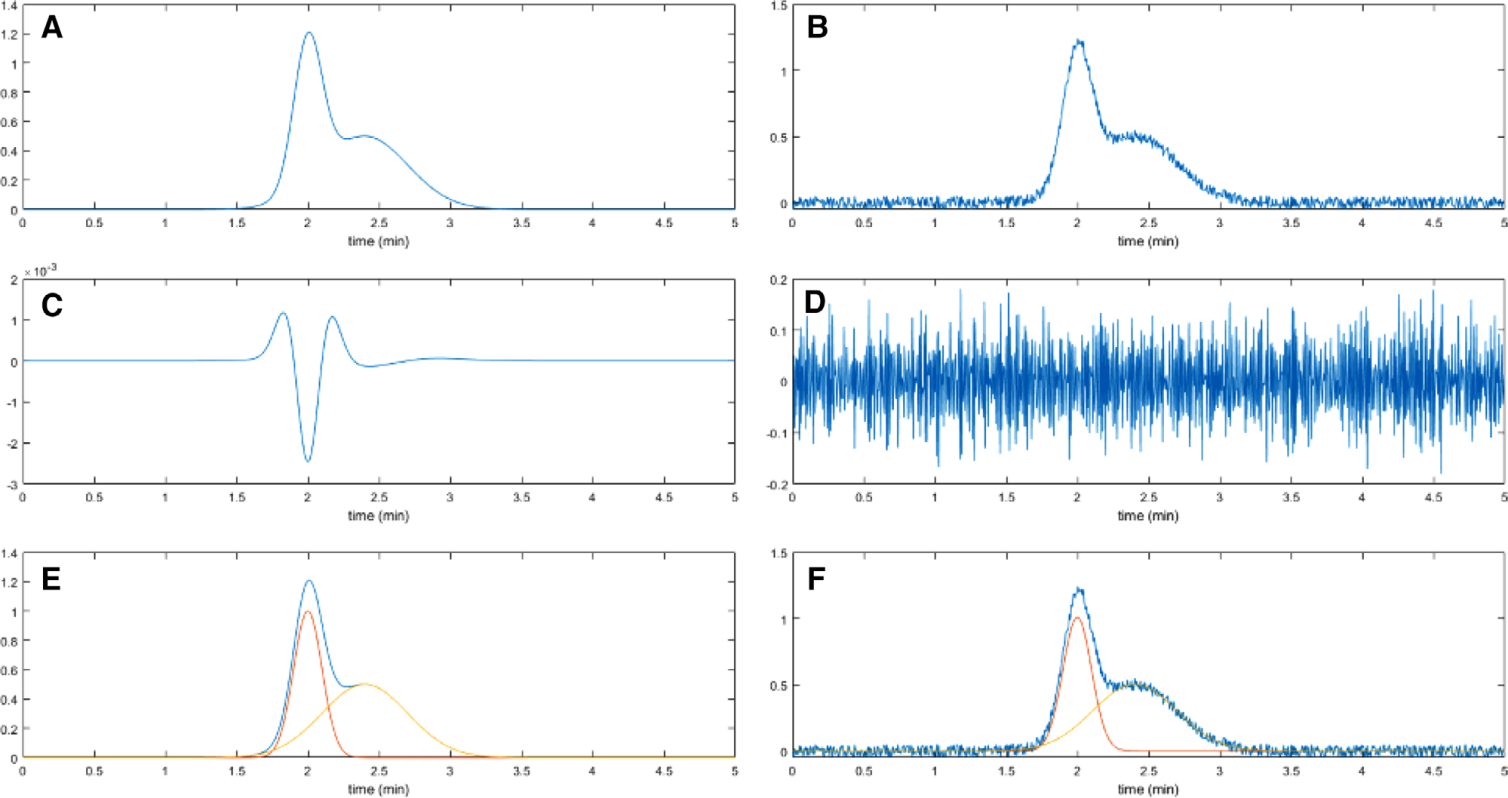
(A) Optimally preprocessed signal of two convoluted peaks; (B) noisy signal of the same two peaks as in (A); (C) second derivative of (A); (D) second derivative of (B); (E) two curves fitted to signal of (A); (F) two curves fitted to signal of (B)

Traditionally there are two primary methods for peak detection. The first approach employs the derivatives of the signal [4, 22, 99]. Taking the derivative will enhance the variation in the original signal [100]. This is illustrated in Figure 8A, where we consider a convoluted chromatographic peak that has undergone perfect preprocessing. The second derivative yields a clear valley at the location of the peak apex (Figure 8B). However, when we regard the case illustrated in Figure 8C and D, the second‐derivative approach appears useless. Indeed, this classical approach is not robust in the presence of noise, system peaks, or other artifacts. It also requires the peaks in question to be sufficiently resolved. The general downside of derivative‐based methods is their sensitivity to noise and the resulting requirement for extensive preprocessing, thus risking a loss of information.

The second approach to peak detection is based on curve fitting or so‐called matched filtering to describe the peaks [101, 102, 103]. Figure 8E and F illustrates that curve‐fitting methods are much less affected by noise. The downside of curve‐fitting methods is that they generally assume the peaks to have perfect Gaussian shapes, which is rarely the case. In some cases, a more flexible peak function is used. This may, however, lead to the detection of non‐existing peaks (false positives) or to overlooking existing peaks (false negatives) [104]. Another downside of the curve‐fitting method is that the deconvolution of heavily overlapping peaks is challenging because the algorithm often cannot determine the correct number of peaks to be fitted. Because perfect methods for peak detection do not exist, numerous research groups are trying to improve the robustness of peak detection and to reduce the number of false positives and false negatives.

#### 3.1.2 Recent developments in peak detection

Separating real peaks from noise is troublesome for some of the peak‐detection methods, such as those based on derivatives. Smoothing strategies rely on the assumption that noise is random and becomes zero when averaged. Consequently, smoothing based approaches can be seen as a combination of data pre‐processing and peak detection. When the noise is removed, the real peaks remain. Besides data averaging, more advanced smoothing‐based methods have been developed for peak detection.

The Gaussian‐smoothing algorithm considers the local maximum point in a section of the chromatogram—as do all smoothing‐based peak‐detection methods. Each local maximum is seen as a peak. Without any preprocessing or additional calculations, noise will result in additional peaks being detected. Actual peaks should retain the local maximum after smoothing, whereas the maxima of noise will disappear when sufficiently large smoothing windows are applied. The Gaussian‐smoothing algorithm of Fu et al. follows three steps [70]. The first step corrects for background drift, the second step is the actual peak detection, and the final step involves peak filtration. By performing the smoothing with different window sizes, the points where maxima disappear under stronger peaks can be determined. The width of the smoothing window is empirically selected.

**FIGURE 9:**
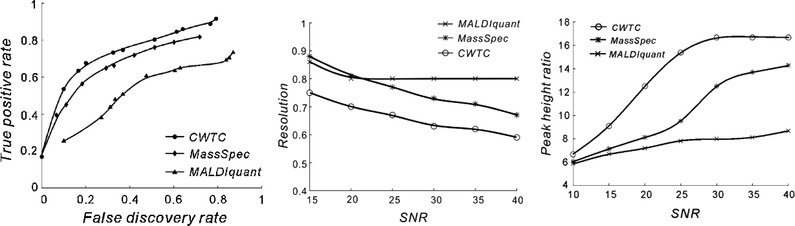
Performance of the CWTC, MassSpec, and MaldiQuant methods tested on ToF mass spectra. Figures show the ratio between true and false positive ratios for the three methods based on simulated MALDI‐TOF data (left), the minimal resolution the algorithm can correctly process given a specific S/N ratio (middle) and the minimal detectable peak‐height ratio, also as a function of the S/N (right). Reproduced with permission from [94]

A popular alternative for the Gaussian‐smoothing algorithm is wavelet‐transform peak detection. The robustness of any fitting‐based method relies on the selection of the number of compounds in a convoluted signal. Peters et al. developed a method based on cross‐validation to determine the optimal number of components [105]. Wavelet transform encompasses the concepts of curve fitting and matched filtering. Curve‐fitting methods generally are known to struggle with a high variability in peak height and width. To overcome these disadvantages, other wavelet shapes have been proposed [106]. An example of a wavelet‐based method is the improved continuous‐wavelet‐transform (CWT) approach, which is thought to handle noisy and overlapping peaks better than alternative techniques (MALDIquant and MassSpecWavelet [107]). This is illustrated in Figure 9.

The CWT approach considers more information on the peak shape, such as symmetry, which reduces the false‐positive rates compared to the traditional derivative [48, 108] and Gaussian‐curve‐fitting [109] methods. CWT can consider more information, because when more complex models are used more properties can be captured, to improve the compatibility with overlapping peaks, CWT has been combined with ridge‐detection algorithms [110]. Such algorithms determine the position of a peak by calculating the local maxima [106]. The downside of CWT methods is that they are non‐numerical, which makes it difficult to determine the area of a peak. The continuous‐wavelet‐transform algorithm of Zheng et al. showed more true positives and fewer false positives than simple smoothing methods and the standard wavelet‐denoising peak‐detection method [107]. Tong et al. developed the CWT approach further, referring to their approach as recursive wavelet peak detection (RWPD). The authors demonstrated that their algorithm performed well for the deconvolution of real data [111]. The RWPD yielded a fit error of 1.2% on simulated data, compared with a 3.2% error obtained with Peakfit, which is a common peak‐fitting tool [112]. By combining the continuous wavelet transformation with heuristic optimization of the peak width, heights, and areas could be determined more accurately.

While the wavelet‐transform methods incorporate more information present in the chromatogram, these methods are also more prone to miss convoluted peaks as compared to Gaussian‐smoothing algorithms [70]. Fu et al. [70] point out a number of problems associated with wavelet‐transform‐based peak detection. However, Yu et al. demonstrated that the Gaussian‐smoothing algorithm had similar problems with convoluted peaks [72].

A recent development in curve‐fitting methodologies has been the introduction of the normal‐gamma‐Bernoulli (NGB) model by Kim et al. [113]. This iteration of a distribution function to describe a chromatographic peak started with a recent open‐access tool, msPeak, which uses the normal‐exponential‐Bernoulli model (NEB) [114]. This approach combines simultaneous removal of noise, baseline correction, and peak‐region detection. Thereafter, peak filtering is performed by fitting different probability models to reduce the number of false positives. Unlike the NEB model, the more flexible NGB model [113] has no analytical solution. However, the authors demonstrated that the newest model fits the data better and leads to more true positives when used to detect MS peaks with low total ion currents [113]. The NGB model found the same true positives in a GC×GC‐TOF data set of 76 compounds and double the number of true positives on MTBSTFA‐derivatized amino acid compounds data compared to the NEB model.

Many peak‐detection methods yield a binary answer (true or false) to the question whether a data point belongs to a peak. As an alternative, the so‐called Bayesian methods focus on probabilities [96]. Originally, this Bayesian approach could not handle overlapping peaks [115]. However, further improvements incorporated the statistical overlap theory [116] and allowed resolving overlapping peaks [117]. The primary advantage of Bayesian approaches over other methods is that they can include prior information. This renders such methods more compatible with experiment with limited number of experiments. A Bayesian method has been successfully used by Adutwum et al. for determining the regions of interest [118]. A Bayesian probabilistic model for untargeted peak detection was developed for LC–MS by Woldegebriel et al. [119]. The advantage of the latter approach was that true peaks could be distinguished from chemical noise without any pre‐processing.

#### 3.1.3 Peak clustering

Peak‐detection methods have been applied to 1D chromatograms for many years [100] and improvements are still being made. An even greater challenge is peak detection in comprehensive 2D chromatography [95]. Data from such experiments can be viewed as a 2D chromatogram or, more commonly, as a series of 1D chromatograms [94]. In the latter case, in order to properly describe the 2D peak, peaks detected in individual second‐dimension chromatograms (“modulations”) need to be clustered. In most approaches, peaks are merged based on a decision tree [4]. In the most recent algorithm, peaks detected in each modulation are clustered based on bidirectional overlap, retention time, and unimodality thresholds [120]. Especially the inclusion of bidirectional overlap improves the accuracy and the compatibly with tailing peaks. Although this approach entailed an improvement in comparison with previous peak‐detection methods in 2D chromatography, the error rate for overlapping peaks was still not satisfactory [115]. Using multichannel data may help to correctly cluster peaks by incorporating additional information. The downside of peak‐clustering methods is their dependence on arbitrary thresholds.

A fundamentally different approach is to view a comprehensive 2D chromatogram as an image, instead of a series of 1D chromatograms. Peak‐detection is then generally carried out using the watershed algorithm, which establishes the boundaries of peaks based on the topology of the surface formed by the signal. An illustrative explanation is that the chromatogram is held upside down and flooded with water until the different peak maxima are no longer separated. This method fails when the modulations do not perfectly align, due to retention‐time variability in the second dimension [121]. However, preprocessing steps may alleviate this issue [94]. The watershed algorithm has recently been applied to clean up GC×GC chromatograms by removing “streaks” [122].

### 3.2 Peak properties

After the peaks are correctly detected, their properties can be determined. These include height, area, and asymmetry. Curve‐fitting methods can determine these properties from the fitted curve, which may be described by rather complex equations. Other methods, such as derivative‐based approaches, require integration.

**FIGURE 10:**
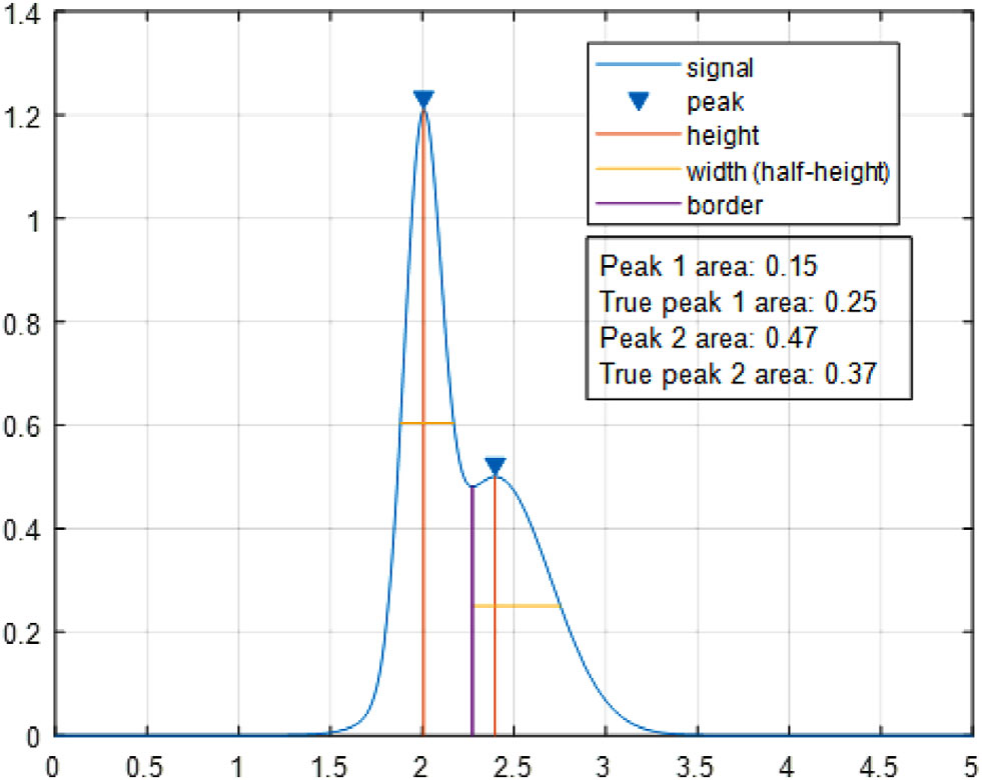
Example of automated peak integration of two convoluted peaks and a comparison of the results with the true values. Peak 1 is on the left; Peak 2 is on the right

Peak integration is often done by standard software that is provided with the hardware (Figure 10). The time boundaries of the individual peaks can be estimated by the system, but are often adjusted manually, which leads to operator‐dependent results. Especially for two‐dimensional chromatograms, this approach is too labor intensive.

An effective way to obtain peak properties by computer‐aided data interpretation is through the computation of statistical moments of a well‐separated or fitted peak [123]. One can distinguish the raw moment (Equation 18), the normalized moment (Equation 19), the centralized moment (Equation 20) and the standardized centralized moment (Equation 21).

(18) $$M_{n}=\int_{-\infty }^{\infty }t^{n}*f\left(t\right)dt$$

(19) $$m_{n}=\frac{M_{n}}{M_{0}}$$

(20) $$\mu_{n}=\frac{\int_{-\infty }^{\infty }t_{rel}^{n}*f\left(t\right)dt}{M_{0}}\;$$

(21) $$\tilde{\mu_{n}}=\frac{\mu_{n}}{\sigma^{n}}$$

The most‐useful moments for determining peak properties and the corresponding equations [124] are stated in Table 1. When curve‐fitting‐based peak detection is used, the f(t)dt part of the raw moment can be replaced by the specific model [125]. The accuracy of the moments may depend on the model used [126]. If there is no fitted model available or if no analytical solution can be found, the peak can be numerically integrated using trapezoidal or Simpson's rules [127]. Next to the number of points per peak, which depends on the sampling frequency of the applied detector [128, 129], the accuracy of the moments also heavily depends on the pre‐processing [130].

**TABLE 1:** Peak moments and their significance

Moment ordinal	Peak property	Formula	Equation
0 (*M*_0_)	Area	$\int_{-\infty }^{\infty }f\left(t\right)dt$	(22)
1 (*m*_1_)	Average time	$\frac{\int_{-\infty }^{\infty }t*f\left(t\right)dt}{\int_{-\infty }^{\infty }f\left(t\right)dt}$	(23)
2$\;(\mu_{2})$	Variance (σ^2^)	$\frac{\int_{-\infty }^{\infty }t_{rel}^{2}*f\left(t\right)dt}{\int_{-\infty }^{\infty }f\left(t\right)dt}$	(24)
3 ($\tilde{\mu_{3}})$	Skewness	$\frac{\int_{-\infty }^{\infty }t_{rel}^{3}*f\left(t\right)dt*\sqrt{\int_{-\infty }^{\infty }f\left(t\right)dt}}{{\left(\int_{-\infty }^{\infty }t_{rel}^{2}*f\left(t\right)dt\right)}^{\frac{3}{2}}}$	(25)
4 ($\tilde{\mu_{4}})$	Kurtosis	$\frac{\int_{-\infty }^{\infty }t_{rel}^{4}*f\left(t\right)dt*\int_{-\infty }^{\infty }f\left(t\right)dt}{{\left(\int_{-\infty }^{\infty }t_{rel}^{2}*f\left(t\right)dt\right)}^{2}}$	(26)

### 3.3 Information extraction

Once the peaks are found and integrated, the interpretation of the obtained results is the next crucial task. Using more‐advanced analytical systems in terms of dimensionality and sampling frequency yields large data sets, from which it is more difficult to extract the relevant information, particularly when samples are complex. To simplify this problem, dimension reduction can be applied to the data. There are many different methods available to extract information. In this review, we address the most popular methods.

**FIGURE 11:**
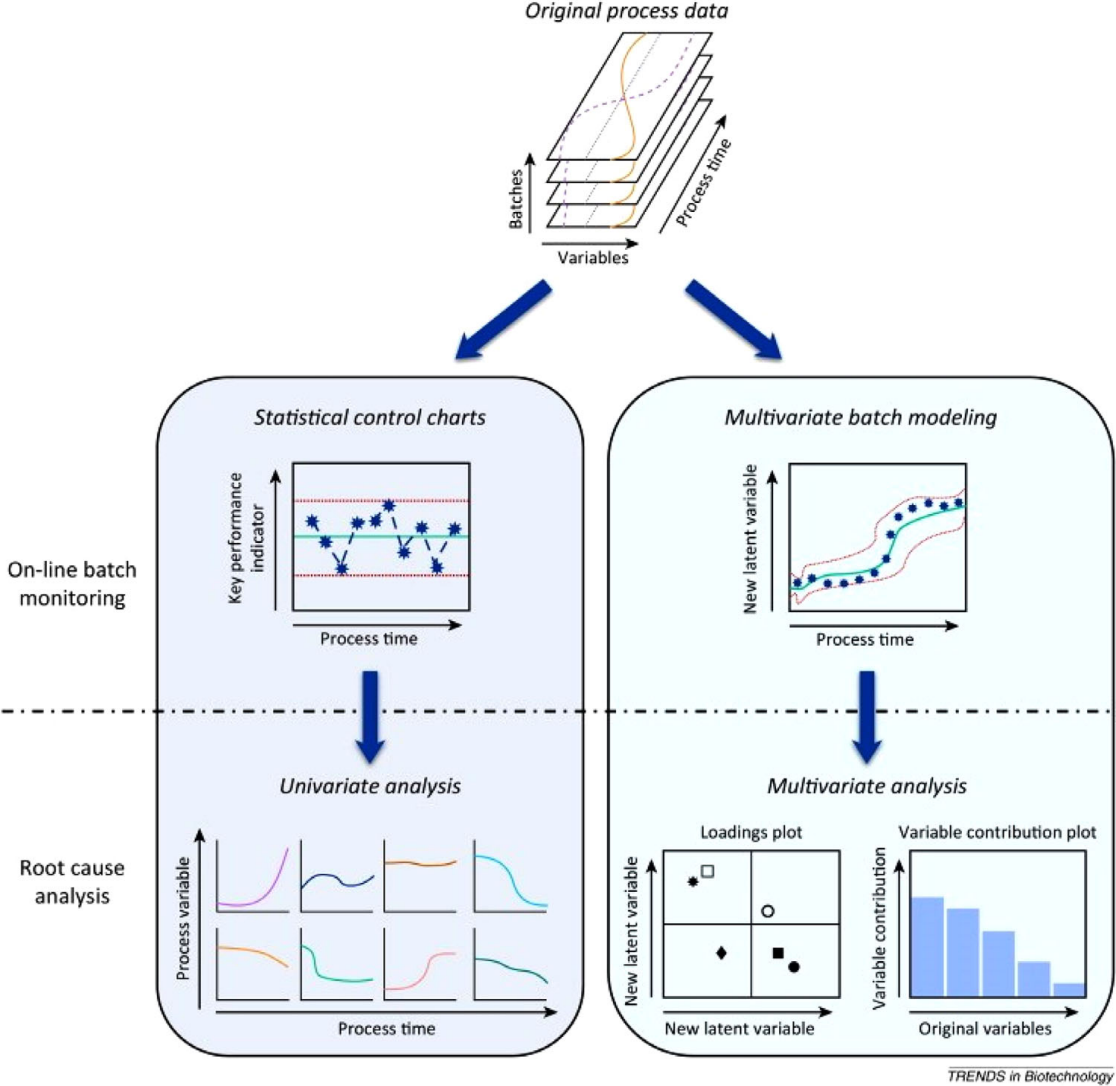
Graphical comparison of univariate (left) and multivariate (right) analysis of bioprocess data. Reproduced with permission from [131]

Univariate statistics describe the variation in a single variable. In multivariate statistical analysis, multiple variables are considered that may be correlated and create a new latent space. Figure 11 presents a graphical representation of univariate and multivariate data analysis as provided by Mercier et al. [131]. Tools for dimension reduction, such as principal component analysis (PCA), can be applied to extract the most informative variables.

#### 3.3.1 Exploratory methods

##### Principal‐component analysis

PCA is often used in chromatography for exploring the results obtained from complex samples [132]. PCA linearly fits uncorrelated variables through the data set. The first component represents the principal variance in the data, the second component explains the second‐most‐occurring variance, and so on. This chemometrics tool is especially useful to aid in the interpretation of high‐dimensional data. As for any chemometrics tool, the results of PCA were greatly affected by the quality of the data (after preprocessing). Prior to PCA, the chromatograms are often first aligned using the COW algorithm [133], since it is regarded as a robust method for alignment [134]. To translate the results to a classifier, which classifies samples into groups based on a provided model, PCA can be combined with a classification method, such as linear discriminant analysis (LDA) [135].

An interesting application of PCA in combination with COW within the field of chromatography is to compare columns [136]. The chromatograms are first aligned with a COW algorithm prior to the PCA, such as to maximize the probability (*p*‐) values. By calculating the Mahalanobis distances and converting these to *p*‐values significant differences between chromatograms can be established.

Binning of data can show improvement of classification by PCA, because the large data set is simplified, removing artifacts and noise. In principle, the bin size is set slightly larger than the width of the peaks in the chromatogram. This results in a dataset in which every component is represented by a single data point. While this approach reduces noise and, therefore, increases the S/N ratio, there is a chance that multiple components are convoluted (so‐called “over‐binning”) and that chemical information is lost. The optimal bin size was reported to depend on the sample [137]. Large bin sizes can be applied when sample compounds are well separated. This approach can be useful if the classification of the samples is important and the raw data are not suitable for direct PCA.

##### Parallel factor analysis

Factor analysis is similar to PCA in that it reduces the dimensionality of the data set. However, where PCA is merely a dimension‐reduction technique, factor analysis also assumes an underlying model and, therefore, finds not only a subspace but also the vector orientations [138]. Parallel factor analysis (PARAFAC) views data as trilinear and containing three modes, *viz*. chromatograms, concentrations, and spectra [139]. Khakimov et al. developed PARAFAC2 [140], which can also deal with small shifts in retention time.

##### Multivariate curve resolution

Apart from being a useful preprocessing tool, MCR can also be used to obtain information from chromatograms. Cook et al. showed that it is quite useful for quantitation [3, 141]. MCR resolves the components of a mixture by deconvoluting the data into response profiles and peak areas. The combination of MCR with ALS (see section 2.4.2) is a useful tool to extract individual chromatographic and spectral profiles for each analyte. In some cases, the resolved spectral profiles may still be noisy [142]. By replacing ALS with an elastic‐net algorithm (ENALS) [143], the tool becomes more compatible with sparse data [142], such as mass spectra. The ENALS algorithm minimizes the number of *m*/*z* peaks when extracting the profiles of an analyte and it eliminates the need for intensity thresholds. By using ENALS, a data reduction by 99.7% was achieved [142], which strongly reduced the computational resources required.

##### Machine learning and deep learning

**FIGURE 12:**
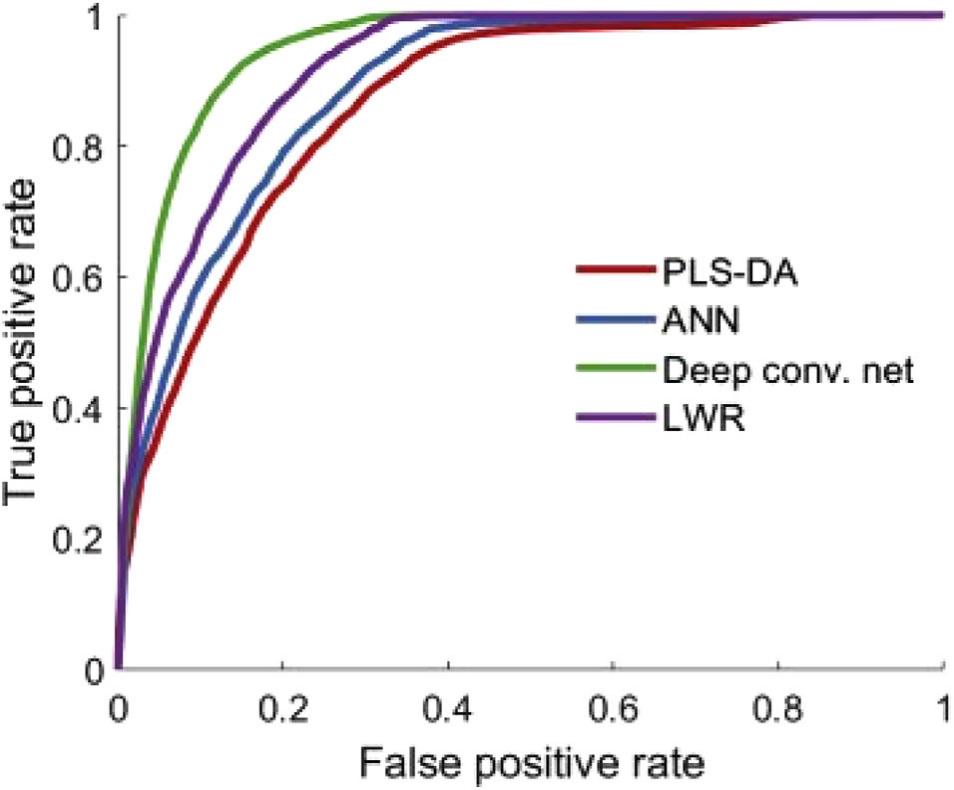
Performance comparison by receiver operating characteristic curves of PLS‐DA, ANN, deep‐convoluted network, and LWR methods, showing the ratio between true and false positive ratios. Reproduced from [145] with permission

Many of the previously described methodologies may be called machine‐learning techniques. Deep learning is a form of machine learning that requires less input from the operator [144]. The more complex the machine‐learning algorithm, the more data it requires for proper training. In deep learning, which uses neural networks, an arbitrary number of layers, possibly with different properties, are used to fit all descriptive relationships in the data. The difference between deep learning and the use of a shallow artificial neural network. So far, there are just few examples of the use of deep learning in chromatography in the literature. Risum et al. used 70 000 elution profiles [145] as input, which were extracted from a GC–MS data set with PARAFAC2. As demonstrated in Figure 12, the deep‐convoluted network performs better than partial‐least‐squares discriminant analysis (PLS‐DA) [145] locally weighted regression (LWR), and a shallow artificial neural network (ANN).

Recent work of Kantz et al. [146] also showed that deep neural networks can remove up to 90% of false‐positive peaks in a nontargeted analysis based on LC–MS data, without losing true positive signals.

### 3.4 Classification

Using chromatographic data, chemometrics can be applied to discriminate between classes of samples with different chemical compositions, such as biological samples. Chemometrics is needed especially when it is a priori unknown which compounds are indicative of a particular class of sample. To discriminate between classes, so‐called untargeted approaches can be used, where a number of chromatograms are used as input data for each class. These can then be used to construct models by which samples can be discriminated. These approaches often involve the reduction of variables to allow robust discrimination between classes. An overview of the most commonly used classification methods and some applications on chromatographic data is given below.

#### 3.4.1 Partial‐least‐squares discriminant analysis

**FIGURE 13:**
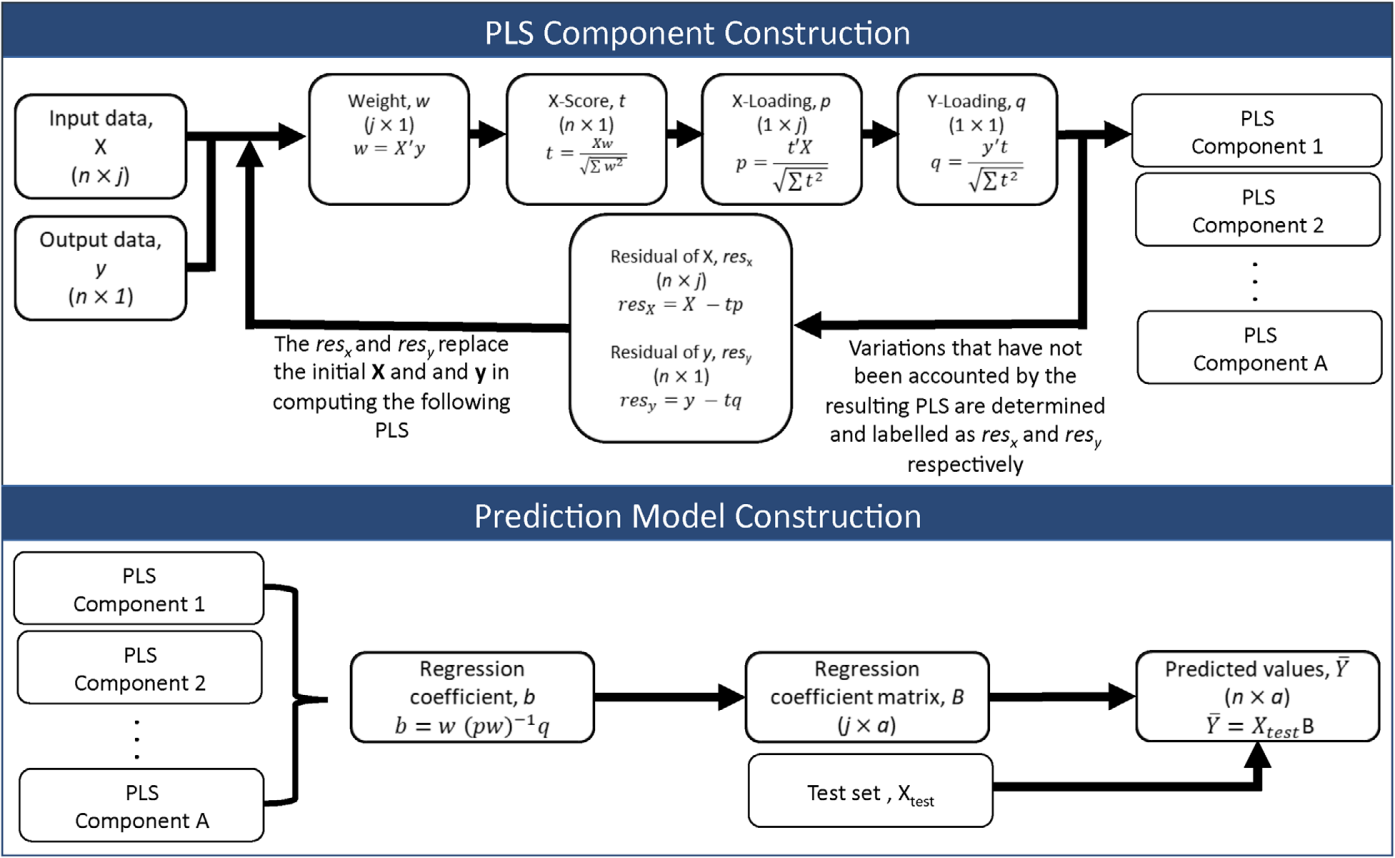
Schematic representation of a PLS1‐DA algorithm, in which *J* is the number of variables, *X*_test_ is the test set, and $res_{x}$ and $res_{y}$ are the *x* and *y* residuals, respectively. *n* is the number of samples. Adapted from [148]

Partial‐least‐squares discriminant analysis (PLS‐DA) can be applied to discriminate classes based on input data and classifiers. PLS‐DA is one of the most‐used chemometrics tools for classification [147]. There are numerous PLS algorithms and adaptations for discriminant analysis, but they share the same basic concepts, which will be briefly explained below. PLS‐DA modeling consists of two main steps, *i.e*. dimension reduction and construction of a predictive model. For multi‐class problems, PLS2‐DA is often applied. In this method, a dummy matrix *Y* is created with dimensions *n* × *g* with *n* being the number of samples and *g* the number of classes [148]. Each class is indicated with binary values in the dummy matrix. The process is depicted in Figure 13. In the first step of the process, the weights are estimated by maximizing the covariance between the input data *X* and the output data *y*. Subsequently, the *X*‐score, *X*‐loading, and *Y*‐loading are determined, and the first component is constructed. Based on the residuals, the subsequent components are determined. Because PLS‐DA is prone to overfitting, the constructed model should always be tested on a subset of the data for verification [148, 149]. Orthogonal‐partial‐least‐squares discriminant analysis (OPLS‐DA) is a variant of PLS‐DA, which is optimized to separate the discriminatory dimension from the non‐discriminatory dimension, yielding results that are easier to interpret [150].

PLS‐DA was applied many times in biomarker‐discovery studies, for example, by Bayci et al. who studied advanced‐stage melanoma in serum with LC–MS/MS and NMR spectroscopy [151]. Dittigen et al. applied PLS‐DA for the classification of black rice based on its geographical origin using LC–MS data [152]. Caldeira et al. applied PLS‐DA on GC× GC–MS data of breath volatiles to differentiate between asthmatic and non‐asthmatic children [153]. Zhang et al. applied OPLS‐DA to verify the authenticity of fruit juices based on a set of 43 metabolites identified by LC–MS/MS [154]. A similar approach was applied by Yuswan et al., who applied OPLS‐DA to LC–MS/MS proteomics data to discriminate between meat samples originating from different organisms [155].

#### 3.4.2 PCA–linear discriminant analysis

PCA is another commonly used tool for classification, although it does not consider a priori classes. For classification purposes, PCA is usually combined with linear discriminant analysis (LDA). Since LDA has the constraint that the number of variables should not exceed the number of samples, it can only be applied to high‐dimensional data after PCA. LDA aims to maximize interclass variation and minimize intraclass variation by creating latent variables that are linear combinations of the original variables [147]. Klockmann et al. applied PLS‐LDA to classify hazelnuts based on geographical origin using UPLC–MS/MS data [156]. Liu et al. applied PCA‐LDA for authenticating wine vintage based on HPLC–DAD data [157].

#### 3.4.3 Soft independent modeling of class analogy

Soft independent modeling of class analogy (SIMCA) is another commonly used classification method. In SIMCA, a model is created by performing PCA on each sample class individually. Each observation in the test set is then compared to the PCA model of each class, and if it falls within the variance of a class, it is assigned to it. This implies that a sample may in principle be assigned to multiple classes – or to none when it fits within none of the PCA models [158]. Perez‐Castaño et al. applied SIMCA and PLS‐DA on normal‐phase‐LC data to classify palm oils based on their geographical origin [159]. Planinc et al. applied SIMCA on LC–MS data to analyze changes in the N‐glycosylation of therapeutic glycoproteins [160].

#### 3.4.4 Support vector machines

Another approach for sample classification is classification based on SVM. SVM is a machine‐learning technique, which can be applied to both regression and classification problems. The method aims to separate the two classes by a hyperplane. The distance between the hyperplane and the closest samples of two classes is maximized to find the optimal separation. If there is no linear solution, the data can be transformed to a higher dimension in order to find a space in which the samples can be separated by a flat plane. To avoid overfitting, a slacking variable can be introduced, allowing a fraction of the training set to be categorized incorrectly [149, 161]. Xi et al. applied SVM to classify edible vegetable oils based on GC–MS data [162]. Fu et al. applied SVM for biomarker screening and classification based on metabolomics data [163]. Reichenbach et al. applied SVMs to LC× LC–DAD data of urine samples, successfully distinguishing between patients before and after bariatric surgery [164].

#### 3.4.5 Random forest

An alternative method is based on random‐forest (RF) models, which take the form of decision trees. In short, the algorithm works by constructing a ‘forest’ of decision trees, which are created from random subsets of features from a subset of samples. By applying a bootstrapping method, a subset of the data is selected for creating the model and another one for testing the prediction. This is repeated many times to grow a forest of decision trees, and the consensus of all grown trees is then used for prediction [165]. In a comparative study of classification methods on a variety of data sets (NMR and MS data), RF was the top performer based on cross‐validation and external validation test cases [147].

#### 3.4.6 Ant‐colony optimization

Another interesting approach to sample discrimination was presented by Kalogiouri et al. [166], who used LC–MS/MS to categorize different varieties of extra‐virgin olive oils. They applied ant‐colony optimization (ACO) to pick features, which would allow good discrimination by PCA and RF. ACO is an optimization algorithm inspired by the foraging behaviour of ants. By releasing artificial agents, referred to as ants, on a dataset with shared memory, referred to as pheromone, the shortest or optimal route can be determined. This can be applied to a set of nodes, in this case MS features, to find the optimal descriptors for a data set. In each iteration each ant picks a certain number of predetermined features. The amount of pheromone the ant encounters along its trail is registered. Paths that score high are more likely to be sampled, optimizing the system with each iteration. Optionally, prior information can be included by assigning weights to data points. It should be noted that the path does not consist of adjacent nodes. A random set of nodes is assigned in the first iteration if no prior information is present. To prevent the algorithm from prematurely converging to a suboptimal point, the overall amount of pheromone may be decreased with each iteration [167]. In each iteration each ant selects *n* nodes from *L* inputs. The probability of each node being selected can be expressed as

(27) $$P_{i}\left(t\right)=\frac{{\left(\tau_{i}\left(t\right)\right)}^{\alpha }\eta_{i}^{\beta }}{\sum_{i}{\left(\tau_{i}\left(t\right)\right)}^{\alpha }\eta_{i}^{\beta }}\;$$

In which $P_{i}\left(t\right)$ is the probability of point *i* to be selected at time *t*, $\tau_{i}$ is the pheromone modifier, adjusted based on the performance of the ants utilizing this point, $\eta_{i}$ represents prior knowledge which can be added to the model. The α and β exponents dictate the relative influence of the pheromone and prior knowledge, respectively. The adjustment of $\tau_{i}$ is calculated for each iteration based on the following equation:

(28) $$\tau_{i}\left(t+1\right)=\rho \cdot \left(\tau_{i}\left(t\right)\right)\;+\;\Delta \;\left(\tau_{i}\left(t\right)\right)$$

where ρ is a constant indicating the decay of pheromone and $\Delta (\tau_{i}\left(t\right))$ is the adjustment made based on the performance of the node. At t= 0, $\Delta (\tau_{i}\left(t\right))$ is zero for all nodes [168]. ACO has been applied in various bioinformatics applications to select features of interest [168, 169, 170].

Readers interested in a performance comparison of various chemometrics classification models are referred to refs. [147] and [171]. Although both studies compare some of the same classification methods, different methods were found to perform best, which illustrates the dependency of these models on data and variables.

### 3.5 Quantification

Chemometrics approaches may also enhance the quantitative capabilities of chromatographic methods. Multivariate curve resolution (MCR) is often applied to quantify overlapping signals based on detected spectra. This is especially challenging when no pure compound spectra are available. The MCR model is based on the following equation

(29) $$X=C\cdot S^{T}+E$$

where *X* represents the raw data, $S^{T}$ is a matrix of the pure spectral images, *C* is the chromatographic profile, and *E* is the residual error. In MCR–alternating least squares (MCR‐ALS), this equation is solved in the following ways:

(30a) $$C=XS\cdot {\left(S^{T}S\right)}^{-1}$$

(30b) $$S^{T}={\left(C^{T}C\right)}^{-1}\cdot C^{T}X$$

An initial value for either $S^{T}$ or *C* is required, which can be estimated from the data [141]. The initial estimates can thus be concentration profiles or spectra. If pure compounds (or pure‐component spectra) are available, then input spectra can be used for a targeted approach. If the approach is untargeted the initial estimate can be made based on the raw data set [172]. Many methods have been developed to obtain this initial estimate, such as simple‐to‐use self‐modeling analysis (SIMPLISMA), orthogonal projection approach (OPA), and key‐set factor analysis (KSFA) [173, 174, 175]. These methods search the most dissimilar spectra in a data set (e.g. LC‐DAD data) and use these as initial estimates. Several constraints can be applied to MCR, such as non‐negativity, unimodality, and predefined spectra or elution profiles [172]. There are numerous applications of MCR‐ALS in 1D chromatography [172]. Hoeylandt et al. for example, applied MCR‐ALS to deconvolute the chemical composition distribution of polymer blends over an SEC separation by applying deconvolution using DAD data [176]. Salvatore et al. applied MCR‐ALS to quantify phenolic compounds in wines to authenticate their protected designation of origin [177].

When applying MCR‐ALS to two‐dimensional data, the application of MCR‐ALS requires some more considerations. Since the data are acquired as a series of 1D chromatograms, from which the 2D chromatogram is reconstructed, some distortions can occur, such as retention time shifts. This has given rise to some discussion as to which method is more suitable for LC× LC data.

**FIGURE 14:**
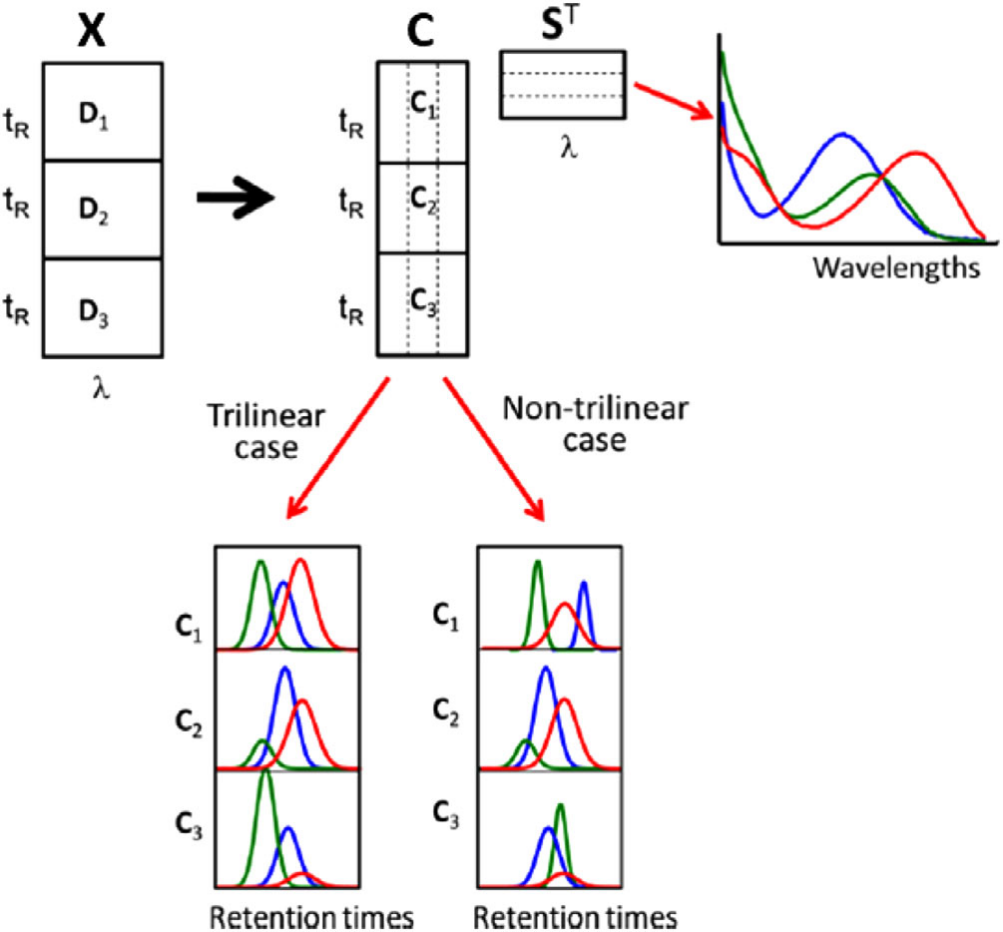
Example showing the difference between a trilinear and a non‐trilinear data set. Reprinted with permission from [178]. Copyright 2012 American Chemical Society

PARAFAC is another method that can be used for quantification, assuming the data is trilinear. Such data can also be analyzed with trilinear variations of MCR‐ALS [178]. The difference between trilinear and a non‐trilinear data set is illustrated in Figure 14. Navarro‐Reig et al. investigated this issue for LC× LC–MS data [179]. Both MCR‐ALS and PARAFAC methods were applied on an LC‐× ‐LC–MS dataset of triacylglycerols (TAGs) in corn oil samples. They found that, due to factors such as retention time shifts and peak shape changes, bilinear models were better suited for LC×LC–MS data than trilinear models. Bilinear MCR‐ALS proved to be the most favorable method. In more‐recent work Izadmanesh et al. compared different MCR‐ALS and PARAFAC models for the analysis of GC× GC–ToF‐MS data of metabolites. They also arrived at the conclusion that MCR‐ALS was most suitable [180]. MCR‐ALS was also applied by Omar et al. for resolving co‐eluting compounds in GC× GC–MS data from *Cannabis sativa* extracts [181]. Another interesting approach utilizing MCR‐ALS‐LC × LC was devised by Rutan et al. The authors developed a novel method for LC × LC‐DAD quantification based on MCR‐ALS. The LC × LC system featured two DAD detectors, one after the first dimension and one after the second. Because more pure spectra were obtained from the second‐dimension detector, improved accuracy in quantification by MCR‐ALS could be achieved [141].

## 4 OPTIMIZATION

### 4.1 Introduction

**FIGURE 15:**
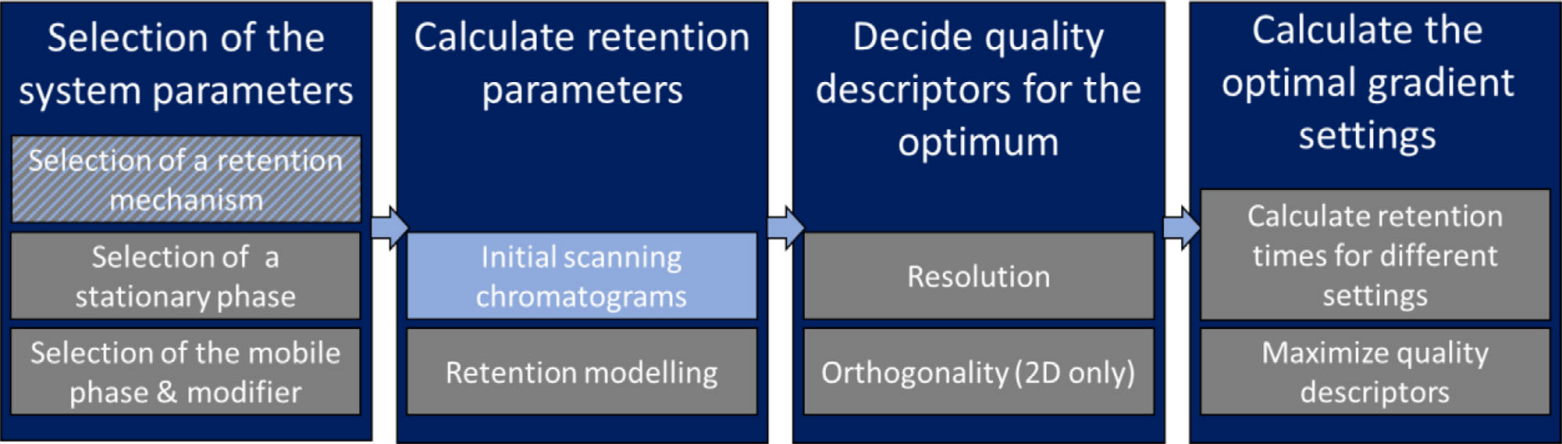
Workflow of optimizing a separation using chemometrics. For the selection of the retention mechanism (Striped box), some knowledge of the sample is required. The scanning chromatograms (Light blue box) are, in principle, the only experimental part of the optimization

Another branch of chemometrics in chromatography concerns the development and optimization of chromatographic methods. In such a process several steps must be taken. Especially, the development of a two‐dimensional chromatographic method can be a cumbersome and challenging task [182, 183]. The sheer number of variables that must be taken into account [184] render a “trial and error” optimization impractical and time‐consuming. Chemometrics tools may aid in almost all of the steps outlined in (Figure 15) [185]. LC method development starts with the selection of system parameters [186, 187] and retention modes (“mechanisms”). The latter usually implies choosing a stationary phase and the constituents of the mobile phase. The selection of the appropriate retention mechanism(s) for a specific separation requires knowledge and expertise of the chromatographer. The chemistry and properties of the analytes and other sample components should to some extent be known. Stationary‐phase and mobile‐phase selection will be discussed in Section 4.2. Once the system parameters are selected, initial scanning chromatograms should be recorded. The results of these can then be used as a starting point for further experiments, to establish retention models for the analytes, or as input for chemometrics modelling methods, such as artificial neural networks (ANNs) (see Section 4.3.1). After retention parameters or ANNs are established, quality descriptors (Section 4.3.2) must be selected. These provide an objective value for the quality of the separation. Examples include peak capacity, resolution, and, for 2D‐chromatography, orthogonality. When quality descriptors have been selected, the system can be optimized by calculating numerous simulated chromatograms under varying conditions.

The optimization step in Figure 15 is not very well defined. A decision as to what and how to optimize highly depends on the aim of the analysis. In targeted optimization method parameters may be modified so as to achieve a specific goal (e.g. peak A separated from peak B, with the resolution between these peaks as the quality descriptor), whereas untargeted optimization generally concerns the maximization of other quality descriptors. As target optimization methods are generally difficult to encompass within an algorithm, this review will focus on the application of chemometrics to untargeted optimization. Target optimization can be seen as a special (usually simpler) case of the general optimization strategy. Readers interested in a more detailed discussion of the rationale behind the different forms of optimization are referred to an earlier work [6].

In this section, recent advances in the field will be explained, including optimization of the system and physical parameters, mobile‐phase composition programs in LC (i.e. gradients) or temperature programs in GC and the limits of optimizing a (2D) chromatogram. In addition to method development and optimization, chemometricians have developed tools to aid method transfer by modeling the effect of column parameters [188] and for transferring to newer and faster LC systems [189, 190]. These method‐transfer tools rely heavily on retention‐time predictions, as discussed in Section 4.3.1.

### 4.2 Method and system setup

The first step in setting up a chromatographic system is the selection of a suitable retention mechanism and a corresponding stationary phase. The selected retention mechanism will define the chemical properties that will govern the separation. When the chemical properties of a sample and its components are known, a decision regarding retention mechanism and column selection can be sensibly made. Column‐selection tools usually rely on large data sets, containing retention times measured on multiple columns. Thus, column selection requires significant knowledge and experience. Especially in 2D chromatography, proper selection of a combination of columns is challenging [191].

**FIGURE 16:**
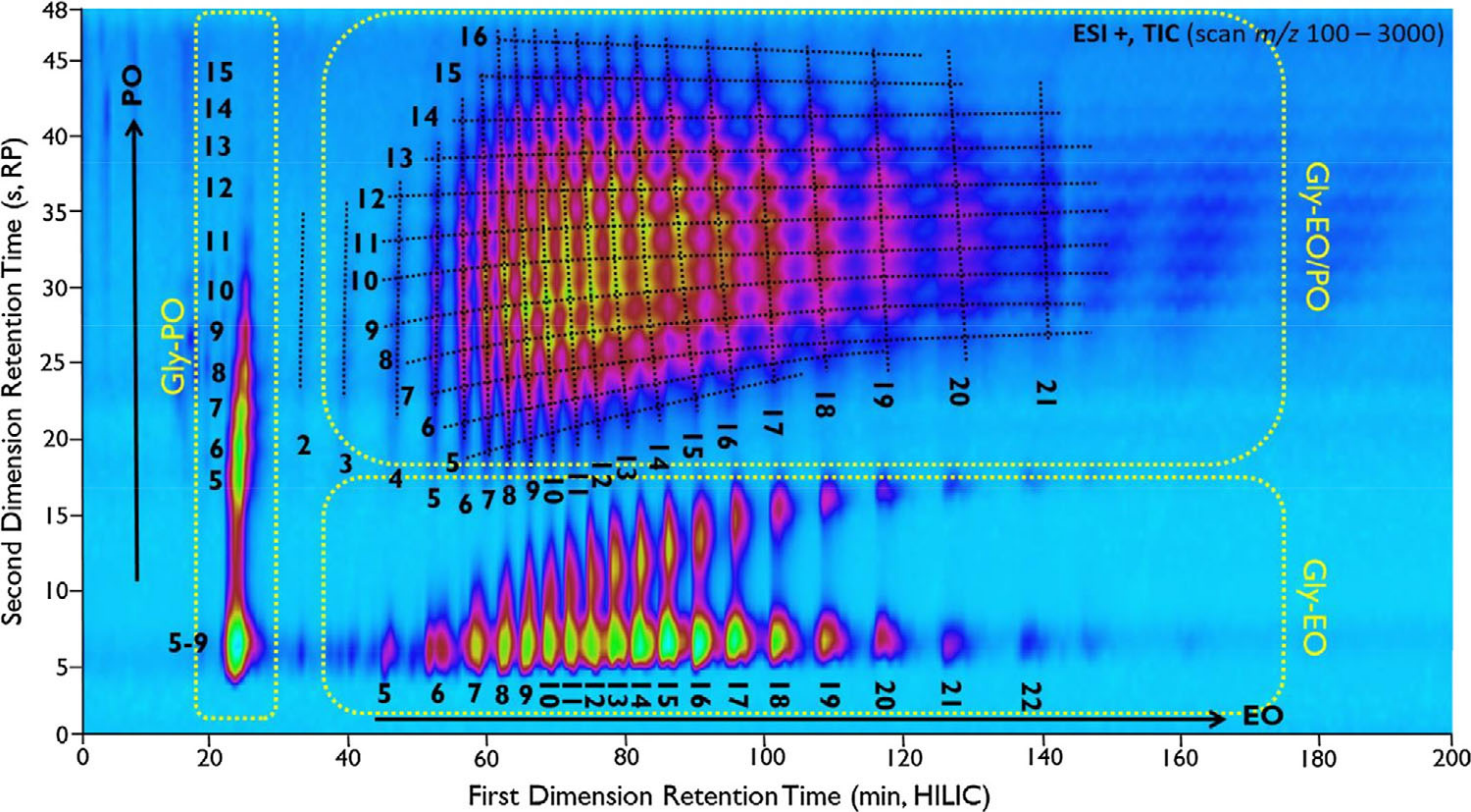
Example of a structured chromatogram. Reproduced with permission from [191]

In GC×GC, the first dimension generally employs a nonpolar stationary phase, which is coupled to a polar stationary phase in the second dimension. An advantage of these polarity‐based systems is that a structured chromatogram can be obtained [192] (Figure 16), which can provide quick insights in the sample. However, maximizing the difference in polarity does not always yield the highest resolving power, as demonstrated, for example, by Seeley et al. [193]. They developed a mathematical model for GC stationary‐phase selection based on the solvation parameters model. The authors analyzed a sample of fatty‐acid methyl esters on 50 different stationary phases. Out of 1225 combinations, the combination of two moderately polar stationary phases was found to provide the best separation.

**FIGURE 17:**
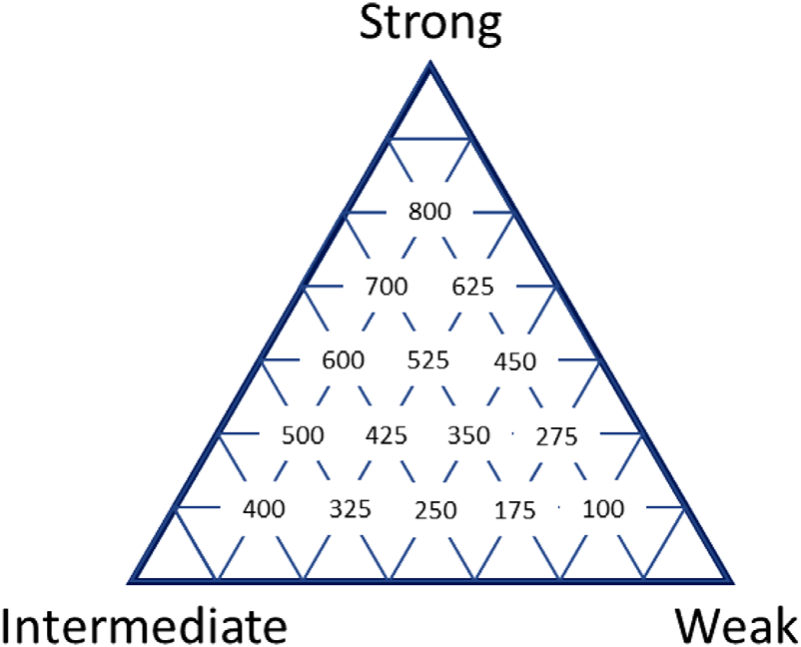
The “PRISMA optimization model.” Each corner represents a different solvent and each point in the triangle represents the solvent strength of the specific solvent mixture. Reproduced with permission from [198]

For LC separations, Euerby, Petersson, and others [194, 195, 196] explored a variety of column parameters, including surface coverage, hydrophobic selectivity, shape selectivity, hydrogen bonding, and ion‐exchange selectivity. The columns were classified in various groups using principle‐component analysis (PCA) and the results can be used as a reference when selecting a column. A similar principle is used in the online “Phase‐Optimization Liquid Chromatography” (POPLC) tool [197] for column selection. This selection tool is based on the “PRISMA‐optimization model” developed by Nyiredy et al. [198]. The PRISMA model is intended as a classification system for mobile phases, where each solvent is classified based on its solvent strength. Different combinations of solvents will yield mixtures with intermediate solvent strengths (Figure 17). Using this concept, the best‐performing solvent combinations can be selected by maximizing the selectivity and resolution of the mixture. With POPLC, stationary phases are classified based on their adsorption strengths, yielding a classification for each column. The databases of Euerby et al. and POPLC are only applicable for reversed‐phase (RP)‐LC. In a more general approach, Krisko et al. suggested a column‐selection method based on several initial runs of a test mixture on an automated column‐switching system [199]. The experimental results were analyzed using DryLab software [200]. Using retention time predictions on all tested columns, the resolution of the mixture components could be predicted. Out of all the predictions, the best‐performing column and gradient program were selected.

In some cases, the required selectivity for a sample cannot be obtained by using a homogeneous column. For this reason, stationary‐phase gradients were recently introduced [201, 202, 203]. However, prediction of retention times on these columns, and, therefore, separation optimization, are problematic. Beer et al. addressed this problem by applying retention modelling for isocratic and gradient separations [204]. Two retention models (Section 4.3.1) by Schoenmakers et al. [205], a log‐linear (“LSS”) model and a quadratic model, were used to predict retention times. Relative prediction errors of 1.60 ± 0.73% were reported. More recently, Jeong and Rutan [206] calculated the rate of migration of each compound per time frame on columns with stationary‐phase gradients, in order to obtain accurate retention‐time predictions (1.94 ± 1.10%), although at the cost of longer computation times.

### 4.3 Selection of analytical method parameters

#### 4.3.1 Optimizing modifier programs

##### Artificial neural networks

After column selection, the temperature gradient for GC or the mobile‐phase composition for LC must be optimized to achieve the best separation possible. In the last decade, artificial neural networks (ANNs) have been explored to calculate retention times for 1D‐GC and 1D‐LC separations [207, 208, 209, 210]. ANNs are computing systems that “learn” to perform tasks by considering many examples. An ANN is able to identify characteristics and trends in data as long as sufficient input is provided. These characteristics and trends are then used to make predictions under new circumstances. ANNs have so far hardly been used for optimizing 2D separations, but recent work in this direction was undertaken by D'Archivio et al. for GC × GC [211]. They used data from Focant et al. [212], who used four different column combinations to separate 209 polychlorinated‐biphenyl (PCB) congeners. Out of these, 70 were used as a training set, with the remaining 139 compounds being used for validation. Single‐response partial‐least‐square (PLS‐1) regression was found to provide the most accurate predictions for the validation set. The same data set was used by Ren et al., who applied a quantitative‐structure‐retention‐relationship (QSRR) model to predict retention times [213]. The compounds were divided into groups with PCA and the “best”‐multi‐linear‐regression (BMLR) method was applied for developing multi‐linear equations. However, in this work the first‐dimension and second‐dimension retention times were not estimated independently. Similar work was performed by Noorizadeh and Noorizadeh, who predicted retention times based on the molecular structure [214]. The authors included 25 compounds in a training set and 44 compounds in a validation set. Multiple QSRR models were tested for retention time predictions. A Levensberg–Marquardt ANN described the retention behaviour most accurately, with a relative error of about 5% in the training set and close to 9% in the validation set.

Although ANNs can provide retention‐time predictions, they require large amounts of data for training. A second argument against ANNs is that, at least in the examples discussed, the molecular structure had to be known to a certain extent. Attempts have been made to apply ANNs for unknown samples [215, 216, 217]. In that case, the models obtained are not related to any physicochemical interactions that occur within the column.

##### Retention modeling

**FIGURE 18:**
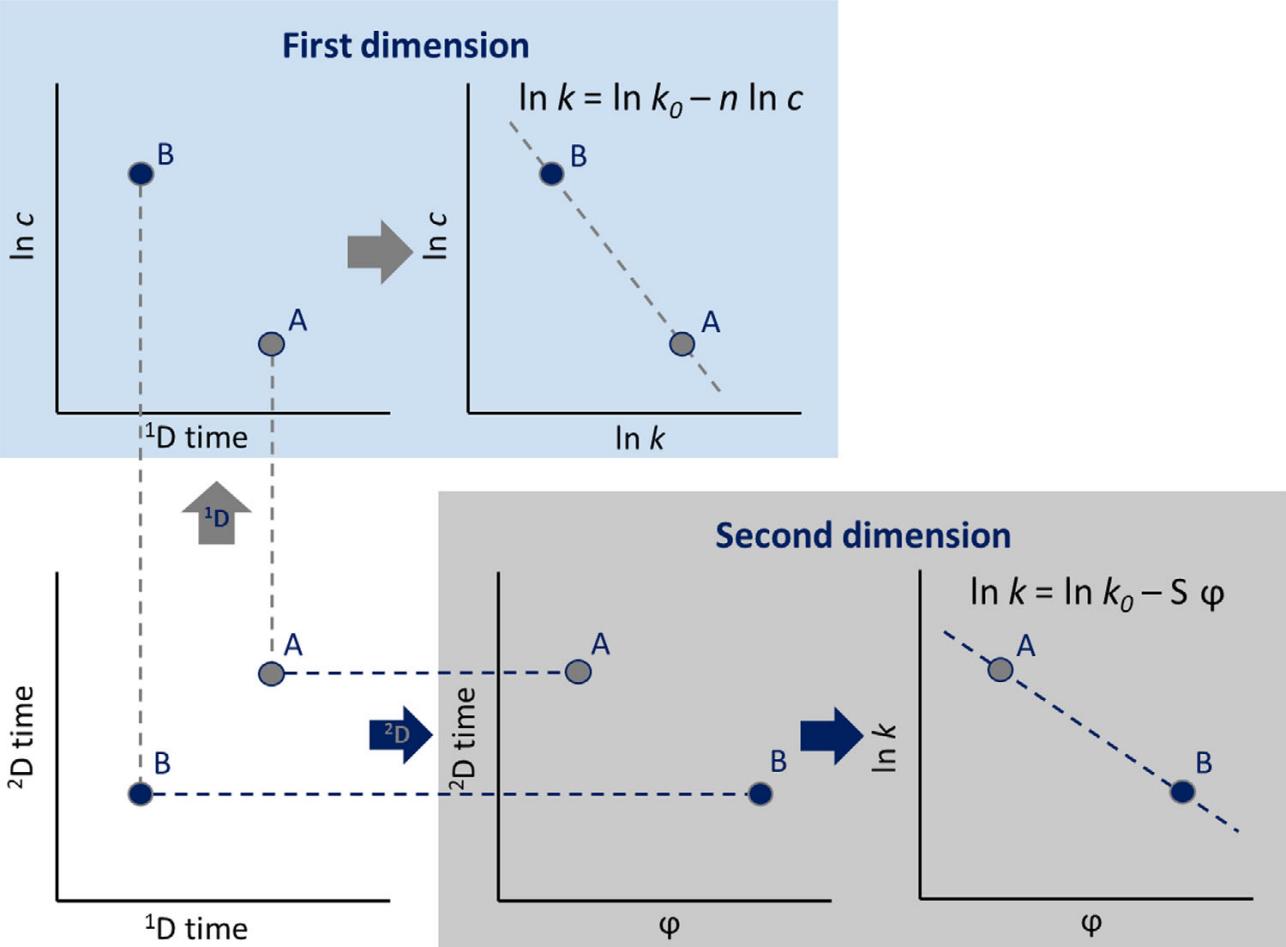
By measuring two (comprehensive 2D) chromatograms using an eluent with a low modifier fraction (A) and a high modifier fraction (B) retention factors can be estimated across a broad range using the appropriate model. Here, the first dimension follows the adsorption model, which is applicable to ion‐exchange LC. The second dimension complies to the LSS (log‐linear) model for reversed‐phase LC. Reproduced with permission from [6]

The classical approach to retention modeling is based on the relationship between retention factor and the physical properties of analytes, stationary phase, and mobile‐phase composition (for LC) or temperature (mainly for GC). These equations usually feature a small number of parameters. Typically, only two or three parameters are required to estimate analyte retention factors obtained across a broad range of different temperatures or mobile‐phase compositions (Figure 18). For GC, retention depends primarily on the stationary‐phase chemistry and column temperature [218]. However, published work indicates more complex relationships between the solute, stationary phase and carrier gas [219, 220]. This has been extended to GC×GC [221, 222, 223, 224].

Retention modeling has been studied extensively for the present variety of retention mechanisms in LC. The most common models are a log‐linear model (often referred to as the linear‐solvent‐strength LSS model) [205], typically used for RPLC; the adsorption model [225, 226], typically used for normal‐phase LC (NPLC) and ion exchange (IEX); the mixed‐mode model [227, 228], which is a combination of the former two models; the quadratic model [205]; and the nonlinear, empirical Neue‐Kuss model [229, 230]. Figure 18 illustrates the simultaneous assessment of retention parameters for two different two‐parameter models, the adsorption model for IEX and the LSS model for RPLC, based on two comprehensive two‐dimensional chromatograms. Although the retention equations are well established for the conventional LC modes (RPLC, NPLC, and IEX), there is still considerable discussion about the most‐suitable (often non‐linear) models for more recent retention mechanisms, such as HILIC [227, 228, 231, 232, 233, 234, 235, 236, 237], SFC [233, 238], and hydrophobic interaction chromatography (HIC) [239, 240, 241].

Strategies for retention‐model selection have been proposed using goodness‐of‐fit tests, such as the Akaike Information Criterion (AIC) [242]. The AIC score is based on the sum of squared errors (SSQ) of predicted versus the real values of a set of *n* datapoints. Adding more parameters in a mathematical model will virtually always result in a better fit, which complicates the selection of the most appropriate model. Therefore, the AIC score also takes the number of parameters (*p*) that are used in the model into account.

(31) $$AIC=2p+n\left(\ln\left(\frac{2\pi *SSQ}{n}\right)+1\right)$$

The AIC proved to be useful for deciding on retention models in HILIC [234, 235, 236]. In very recent work by Roca et al. [243] the statistical F‐test was used, in addition to the AIC, to evaluate the significance of adding an additional parameter to a retention model [236]. The F‐test uses a probability function to compare the $SSQ_{full}$ of a full model and the $SSQ_{red}$ of a reduced model, in which one or more parameters are discarded.

(32) $$F=\frac{{\mathrm{MS}}_{\mathrm{diff}}}{{\mathrm{MS}}_{\mathrm{full}}}=\frac{\left({\mathrm{SSQ}}_{\mathrm{full}}-{\mathrm{SSQ}}_{\mathrm{red}}\right)/\left({\mathrm{df}}_{\mathrm{red}}-{\mathrm{df}}_{\mathrm{full}}\right)}{{\mathrm{SSQ}}_{\mathrm{full}}/{\mathrm{df}}_{\mathrm{full}}}$$

${\mathrm{MS}}_{\mathrm{full}}$ and ${\mathrm{MS}}_{\mathrm{diff}}$ represent the mean squared error of the full model and the mean of the difference in SSQ of the two functions, respectively, and ${\mathrm{df}}_{\mathrm{full}}$ and ${\mathrm{df}}_{\mathrm{red}}$ are the degrees of freedom for the full and the reduced model. The probability (*p*) of the significance of the missing parameters are assessed with the cumulative distribution function of the F‐test. Within a confidence interval of 5% (*p* < 0.05), Roca et al. concluded that the extra terms in the mixed‐mode and quadratic model were statistically insignificant when compared to the LSS and adsorption models when using retention modeling in HILIC.

##### Linear‐free‐energy relationships

Next to retention modeling, linear‐free‐energy‐relationships (LFER) or linear‐free‐solvation relationships (LSER) can be used for retention‐time predictions and for classification of column selectivity [244]. The LFER model describes a solute property in a given system, SP, as the summation of different solute‐solvent interactions. The equation distinguishes between solute parameters (capital letters) and the solvent or system parameters (lower‐case letters). The different descriptors are a system constant *c*, a descriptor related to the polarizability of π‐ and *n*‐electrons (*e*, *E*), the polarity of bond dipoles an induced dipoles (*s*, *S*), acidity (*a*, *A*), basicity (*b*, *B*), and the molar volume (*v*, *V*).

(33) SP=c+eE+sS+aA+bB+vV

When the parameters are known for solute and system, the solute property (e.g. retention time) can be predicted using the above relation. To reliably estimate the parameters, a large number of measurements under different conditions must be performed. Abraham et al. utilized 18 to 613 different measurements for their classification [244]. Ulrich et al. created a database with LFER parameters, called “UFZ‐LSER database” [245], which can in principle be used for retention‐time predictions. Coefficients are regularly being updated. An example is the recent classification of the sorption of an organic compound on carbon black by Su et al. [246]. Ortak and Demiralay [247] recently combined LFER predictions with van‘t Hoff plots to calculate temperature dependencies of retention. For more information, the interested reader is referred to a review on LFER by Endo and Goss from 2014 [248].

#### 4.3.2 Quality descriptors

##### Orthogonality

**FIGURE 19:**
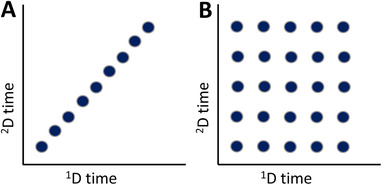
(A) Non‐orthogonal 2D separation methods. (B) Fully orthogonal methods

For an optimal separation of a sample using 2D chromatography, it is crucial that both dimensions have different selectivities [249]. If the selectivities in both dimensions are the same, the separation will take place along a diagonal line across the separation space (Figure 19A). When selectivities differ greatly, more of the separation space will be used (Figure 19B). The degree of dissimilarity is called the orthogonality.

A series of different methods to quantify orthogonality has been developed. Examples include information theory [250], convex‐hull strategies [251], bin‐counting approaches [252], home‐range theory [253], conditional entropy [254], and nearest‐neighbour distances [255]. Comparative studies were conducted by Gilar et al. [256] and later by Schure and Davis [257]. In these studies, the authors described the advantages and disadvantages of each orthogonality metric and concluded that a product of several different orthogonality metrics provided the most robust descriptor. The more recent asterisk orthogonality metric by Camenzuli and Schoenmakers [258] and the modeling approach by Zeng et al. [259] were, however, not included in those studies,. Some older methods, such as the geometric approach of Liu et al. [260] were also excluded. The asterisk metric was tested against many of the existing orthogonality metrics by its creators and was found to be more robust than other metrics [258]. The authors found that the asterisk metric is less affected by a change in the number of compounds, unlike, for example, the bin‐counting approaches, and that its value is less affected by outliers than the convex‐hull strategies. More recently, new metrics were developed by Mani‐Varnosfaderani and Ghaemmaghami, based on the maximal information coefficient [261], and by Leonhardt et al. [262], based on a combination of bin‐counting and calculated histograms for the respective dimensions. Mommers and Van der Waals [263] developed two new metrics based on a polynomial fit and a new bin‐counting approach. Since the developed equations were usually only tested against one or a few other approaches, there is no definitive conclusion on the best approach calculating orthogonality. A product of different approaches, as suggested by Schure and Davis [257], may provide the most robust quantification for orthogonality as shown by Cuzuel et al. [264]. The latter authors used nine criteria, including six different orthogonality metrics to optimize their GC×GC separations. They calculated the “global desirability” as a product of all nine criteria for different experimental set‐ups. The experimental setup with the highest global desirability was deemed optimal. The authors claimed to have established a simple, but highly flexible approach for assessing the optimum, since desirability factors could be added or discarded according to the user's preferences. Similarly, Bassanese et al. described protocols to find the most orthogonal LC×LC separations for their separations [265]. In addition to 2D separations, orthogonality is an important quality descriptor for higher‐order separations. When more dimensions are added to a chromatographic system, the probability of similarities in selectivity will increase. Schure and Davis provided a quantification method for orthogonality in three or multidimensional separation mechanisms [266].

##### Resolution

**FIGURE 20:**
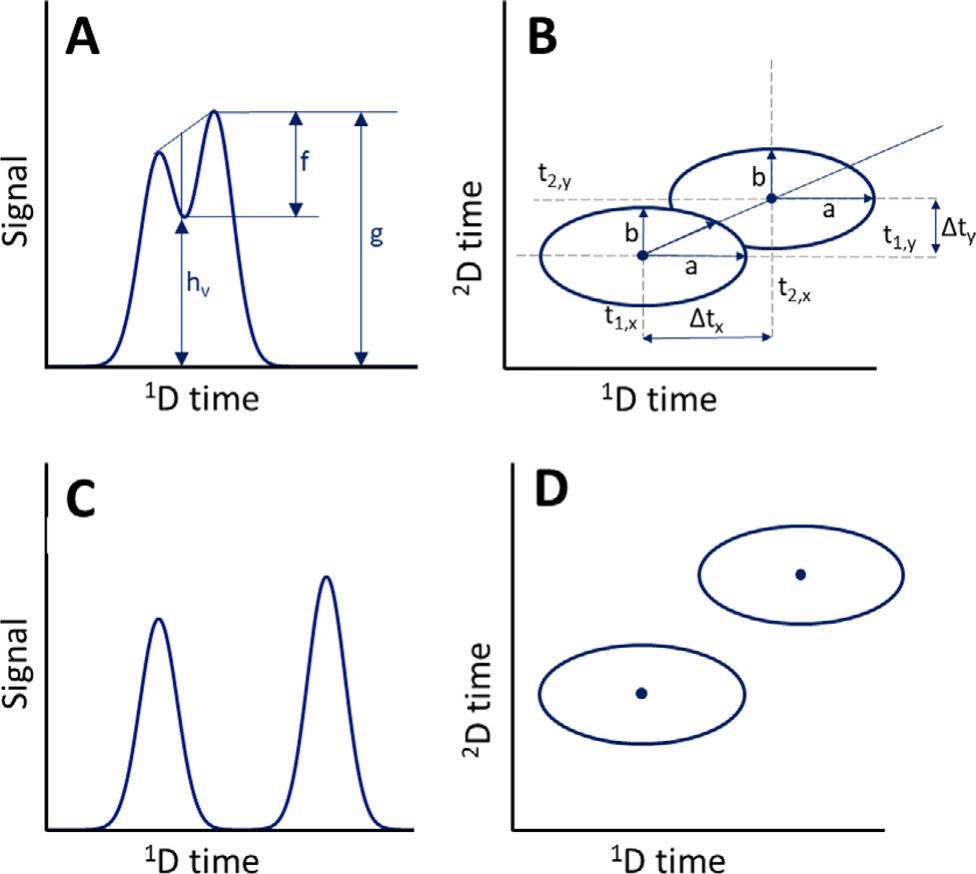
Top: Low resolution, overlapping peaks. Bottom: high resolution, baseline separated peaks. Reproduced from [268] with permission

Resolution in chromatography quantifies the separation between two peaks. In a 1D chromatogram, the resolution ($R_{s}$) can be calculated from the following equation

(34) $$R_{S}=\frac{t_{R,2}-t_{R,1}}{2\sigma_{1}+2\sigma_{2}}\;\approx \frac{\Delta t_{R}}{4\sigma_{\mathrm{avg}}}$$

with $t_{R,1}$ and $t_{R,2}$ the retention times of two adjacent chromatographic peaks and σ_1_ and σ_2_ the corresponding peak widths. A resolution value of 0 represents two completely overlapping peaks with identical retention times, whereas a value of 1.5 or higher corresponds to baseline separated peaks (Figure 20C). The resolution between two chromatographic peaks can also be expressed using the valley‐to‐peak ratio, *P* [267].

(35) $$R_{s}=\sqrt{-\frac{1}{2}\ln\left(\frac{1-P}{2}\right)}\;$$

where *P* is expressed as

(36) $$P=\frac{f}{g}=1-2e^{-\frac{1}{2}{\left(\frac{\Delta t}{2\sigma }\right)}^{2}}$$

The parameters *f* and *g* are shown schematically in Figure 20A. The parameter *g* represents the average peak maximum and the depth of the valley *f* is equal to the difference between this average maximum and the signal height in the valley $h_{v}$. In Eqn. 36 it is assumed that the SD, σ, is the same for both peaks and that both peaks are Gaussian shaped. The above equation can be expanded to multi‐dimensional separations as shown by Schure [268]. The resolution $R_{s}$ is calculated from the same equation (Eqn. 35). Because of the identical equation, Schure concluded that this equation can be used for any number of dimensions. The equation for the peak‐to‐valley ratio *P* can be expanded to incorporate the retention times and peak widths of the extra dimension. The equation for two dimensions is shown below, where $t_{x}$, $\sigma_{x}$, $t_{y,}$ and $\sigma_{y}$ represent the retention time and SD in the first dimension (*x*) and second dimension (*y*), respectively.

(37) $$P=\frac{f}{g}=1-2e^{-\frac{1}{2}{\left(\frac{\Delta t_{x}}{2\sigma_{x}}\right)}^{2}}\cdot e^{-\frac{1}{2}{\left(\frac{\Delta t_{y}}{2\sigma_{y}}\right)}^{2}\;}$$

Peters et al. adjusted the equation formulated by Schure [269]. The authors observed that chromatographic peaks are usually not Gaussian shaped. Therefore, their proposed method does not start from the center of the peaks, but rather from the closest points between them. Furthermore, they proposed a method to determine whether two peaks are neighbors. Thanks to this algorithm, the computational time can be reduced, since the resolution does not need to be calculated between peaks that are not close to each other.

##### Peak capacity

The peak capacity is the theoretical number of peaks that can be resolved under specific conditions and within a certain analysis time. Giddings derived a formula for estimating the peak capacity (*n_p_*) under non‐programmed (isocratic, isothermal) conditions in 1967 [270]. Giddings’ equation features time (*t*), desired resolution (*R_s_*), and the separation power of the column (plate number, *N*_col_).

(38) $$n_{p}=1+\frac{\sqrt{N_{\mathrm{col}}}}{4R_{s}}\ln\left(\frac{t_{R,n}}{t_{R,1}}\right)$$

where $t_{R,\;1}$ and $t_{R,n}$ are the retention times of the first‐ and last‐eluting compounds, respectively. In theory, adding a second dimension to a system will result in a total peak capacity (*n*_2D_) that is the product of the peak capacities of the individual dimensions (^1^ *n* and ^2^ *n*, respectively).

(39) $$n_{2D}{=}^{1}\;n\cdot^{2}n$$

The effective (useful) peak capacity is lower in reality if the two systems are not fully orthogonal. Grushka demonstrated Giddings’ equation to be analytically correct [271]. However, especially in LC, there is a difference in the observed plate number (*N*_obs_) and the column plate number (*N*_col_). Both Giddings and Grushka assumed that virtually all band broadening occurred in the column and that the plate number was identical for all analytes. With sophisticated contemporary columns and stationary phases, these assumptions are often no longer valid. Extra‐column band broadening has a greater impact on the resulting separation when using more efficient columns [272]. Especially in 2D chromatography such extra‐column effects, including those arising from transferring a peak from the first to the second dimension, can significantly affect the peak capacity of a 2D system. The modulation time will decrease the effective peak capacity of the first dimension ($n_{1D}$) due to undersampling. However, a trade‐off arises, because minimizing the modulation time to increase $n_{1D}$ inevitably decreases the resolving power and the peak capacity of the second dimension ($n_{2D}$).

Vivó‐Truyols et al. estimated the loss in theoretical peak capacity in isocratic and gradient LC and estimated a loss of 50% in peak capacity in each dimension, and thus a 75% loss in total [187]. Utilizing a Pareto‐optimality approach [273], Vivó‐Truyols et al. also concluded that two to three cuts per first‐dimension peak would result in the highest observed peak capacity (*n*_2D_). An additional conclusion was that gradient elution provided a significant improvement in peak capacity in comparison with isocratic elution. Potts and Carr confirmed the estimates of Vivó‐Truyols by deriving an exact equation for peak capacity using isocratic elution [274]. Recently, Chester [275] rewrote the equation of Potts and Carr in such a way as to provide insight into the effects of extra‐column band broadening. The equation for effective peak capacity with isocratic elution in each individual dimension then reads

(40) $$n=1+\frac{\sqrt{N_{col}}}{4R_{s}}\ln\left(\frac{t_{R,n}+\sqrt{{t_{R,n}}^{2}+{\sigma_{ex}}^{2}N_{col}}}{t_{R,1}+\sqrt{{t_{R,1}}^{2}+{\sigma_{ex}}^{2}N_{col}}}\right)$$

where $\sigma_{ex}$ is the extra variance due to an imperfect system. In the first dimension, the extra variance is almost equal to the variance added by under‐sampling. In the second dimension, most of the extra variance is due to the large injection volume.

##### Chromatographic response functions

Recently, new chromatographic response functions (CRFs, or chromatographic objective functions, COFs) were developed. CRFs are single‐number descriptors that describe the quality of separation. Most CRFs are based on the number of observed peaks and the time required to obtain the separation. Tyteca and Desmet conducted a comparison study on the performance of multiple CRFs [232], describing the advantages and disadvantages of almost 40 different functions. New CRFs are still being proposed, for example by Alvarez‐Segura et al. [276], who used peak prominences. The latter method was evaluated for complex chromatograms with unknowns or without standards by selecting a global value [277]. the authors concluded that the sum of the COFs provided the most‐robust global value. The product of the quality descriptors was found to be too sensitive to one or more poorly resolved peaks, even though it performed excellently for the remainder of the separation. Duarte et al. extended CRFs to 2D chromatography [99], providing a single value for the quality of a 2D separation based on the number of observed peaks, the analysis time, and an estimate of peak overlap. Nowik et al. combined their orthogonality metric with a function of the desired resolution to create their CRF for 2D separation systems [278]. The calculated values of a CRF can be useful for quickly assessing the quality of a separation. Interested readers are referred to a review on CRFs by Matos et al. [279].

#### 4.3.3 Gradient optimization

Using retention modeling, the retention times of compounds can be rapidly predicted. From the simulated chromatograms produced, an “optimal” result can be predicted using the above‐discussed quality describers, which may then be verified experimentally. Since measuring a 2D chromatogram requires up to several hours and a series of predictions take seconds or minute, automatic simulation and optimization of 2D chromatograms seems very attractive. An additional benefit of this predictive approach is a reduction in the consumption of organic solvents or carrier gases. In GC, adjusting the temperature program and the flow rate may improve the separation. Computer modeling to reduce the method‐development time for GC×GC has been discussed by Dorman et al. [280]. Optimization tools are even more useful in LC, since the elution order may change depending on the gradient program [281].

**FIGURE 21:**
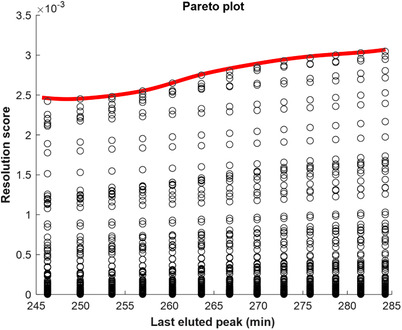
Example of a Pareto plot. The red line depicts the Pareto optimal front

For LC several optimization tools and algorithms have been published. The 1D optimization tool DryLab [200] had already been developed in 1989. Before and after, other optimization methods, such as predictive elution‐window shifting and stretching (PEWS) [231] and “concentration pulses” have been developed. A concentration pulse is a temporary increase in modifier content when a compound elutes and is typically used in multistep isocratic measurements [282] and multistep gradients [283, 284]. These optimization strategies may require a good deal of computational power to perform brute‐force computations. Therefore, root‐finding methods were investigated for reducing the computation time required to locate optimal methods [285]. The concepts used for 1D optimization were recently expanded to LC×LC with the development of the program for interpretive optimization of 2D resolution (PIOTR) by Pirok et al. [286]. The PIOTR approach relies on retention modeling and simulations to rapidly distil optimal method parameters for the first‐ and second‐dimension gradient systems. The desired optimum can be identified using Pareto plots of, for example, resolution versus analysis time (Figure 21). The Pareto‐optimal front depicts a line of optimal conditions. No other point in the Pareto plot will provide a better resolution in a shorter analysis time. Muller et al. [11] recently published methods for the kinetic optimization of HILIC × RPLC separations. They considered not only modifier gradients, but also the system settings, such as column length, temperature, dilution factor, flowrate, and the maximum pressure.

Although the benefits of retention modeling are described in numerous papers, there is still discussion about how to correctly calculate retention times. Blumberg has argued that migration equations outperform elution equations in column‐based separations in which the pressure is kept constant [287]. Because the viscosity changes with modifier composition, constant‐pressure operation applies variations in the flow rate. Conventional retention models do not account for this. Moreover, non‐linear retention models cannot easily be integrated mathematically, with the exception of the Neue‐Kuss model [229, 230], which has been specifically designed to allow integration. However, it is an empirical model which bears no relation to interactions occurring in the column. Numerical integration is used to deal with other non‐linear models [285]. Retention models do not take the injection volume and initial solvent composition into account. These initial conditions are important when transferring compounds from the first to the second dimension, as these conditions influence band broadening. Jeong, Stoll, and others addressed these issues in a series of papers [288, 289], with calculations being performed for each time frame. The authors admitted that their algorithms were slower than conventional modeling, but their predictions were thought to be more accurate.

### 4.4 Peak tracking and alignment

A drawback of retention modeling for optimization of separations is that retention times must be assigned for each analyte of interest in each chromatogram. The interactions between the analyte, stationary phase and mobile phase cannot be modeled without experimental input data. Especially for complex mixtures consisting of hundreds of compounds, it can be a challenging task to assign retention times to all analyte peaks. Accurate automatic peak detection and, possibly, deconvolution of overlapping peaks are required before peaks can be “tracked” or labeled (but not necessarily be identified). Several peak‐tracking algorithms have been developed for LC‐DAD data, such as those of Round et al. [290] and Bogomolov and McBrien [291]. However, hyphenation of LC to MS often is needed to properly track peaks. Several peak‐tracking algorithms have been published for LC–MS data in recent years [292, 293, 294]. For 2D separations, examples of peak tracking have so far been limited to GC×GC data. One example is the algorithm of Barcaru et al. [295], which is based on the use of Bayesian statistics and neighboring peaks. However, as stated earlier, shifts in elution order occur more often in LC and, thus, pairing peaks based on its neighbours is prone to mismatching in LC×LC. Reichenbach et al. published a peak‐alignment algorithm for LC×LC [57] where a presumed pattern of chromatographic peaks and the corresponding metadata, e.g. UV spectra, are identified on one or more initial chromatograms. After the establishing this pattern, new sets of chromatographic data can be compared. The authors published peak‐alignment results varying from 89 to 100% correctly matched peaks. The algorithm allows for small deviations in retention time to account for variations in chromatographic conditions (e.g. column aging or temperature deviations).

### 4.5 Limits to optimization

Irrespective of the described tools and algorithms, there are limits to the optimization of 2D chromatography. Vanhoutte et al. evaluated the separation limits of fully comprehensive LC×LC [296] using a Pareto‐optimality approach. Their work suggested that for relatively simple samples 1D‐LC may outperform LC×LC in terms of analysis time and the maximum number of resolved peaks. However, when sample complexity increases, LC×LC will virtually always outperform 1D‐LC. Nonetheless, excessive under‐sampling of the first‐dimension separation and sample dilution may limit the separation power of LC×LC. Stop‐flow LC×LC, active modulation, or spatial 2D‐LC may alleviate these hurdles [296]. Davis and Stoll have discussed the probability of resolving all peaks in 1D‐LC, fully comprehensive LC×LC, and selective comprehensive 2D‐LC separations (sLC×LC) [297]. They concluded that sLC×LC offered the highest probability of resolving all peaks in complex samples. Indeed, all of these developments concern LC×LC where the availability of vastly different selectivities leaves much room for optimization of selectivity. Instead, method development in GC×GC involves maximizing efficiency (i.e. plate numbers and thus peak capacity), rather than selectivity [182]. Instead, improvement of GC×GC methods is often sought in modulation strategies [298, 299].

## 5 REFERENCE TABLE

Data analysis is a large field and it may take a significant effort to find relevant research. To help the reader get acquainted with the different subjects, some relevant papers are summarized per category as a starting point in Table 2. While more papers on the different topics can be found, the table gives a mostly comprehensive view of recent developments.

**TABLE 2:** Overview of recent and useful applications of chemometrics in chromatography

Background correction			
Title	Subcategory	Year	Reference
Trilinear decomposition method applied to removal of 3D background drift	ATLD	2007	[60]
Leveraging probabilistic peak detection to estimate baseline drift in complex chromatographic samples.	Bayesian statistics	2016	[26]
A concise iterative method using the Bezier technique for baseline construction	Corner cutting	2015	[25]
Assisted baseline subtraction in complex chromatograms using the BEADS algorithm	Smoothing/Filtering	2017	[52]
Background correction in separation techniques hyphenated to high‐resolution mass spectrometry – Thorough correction with mass spectrometry scans recorded as profile spectra	MS Profile spectra‐ based correction	2017	[55]
Simple automatic strategy for background drift correction in chromatographic data analysis	Local minimum values	2016	[46]
Chemometric strategy for automatic chromatographic peak detection and background drift correction in chromatographic data	Derivative‐based peak detection	2014	[48]
Chromatogram baseline estimation and denoising using sparsity (BEADS)	Smoothing/Filtering	2014	[51]
Effect of background correction on peak detection and quantification in online comprehensive two‐dimensional LC‐DAD	SVD	2012	[36]
A fully automated iterative moving averaging (AIMA) technique for baseline correction	Moving‐average smoothing	2011	[43]
An intelligent background‐correction algorithm for highly fluorescent samples in Raman spectroscopy	Smoothing	2010	[31]
Automated autofluorescence background subtraction algorithm for biomedical Raman spectroscopy	Curve fitting	2007	[300]
A new general‐purpose fully automatic baseline‐correction procedure for 1D and 2D NMR data	Wavelet transform	2006	[30]
Baseline correction of spectra in Fourier transform infrared: Interactive drawing with Bézier curves	Bezier smoothing	1998	[42]
A general baseline‐recognition and baseline‐flattening algorithm	Curve fitting	1977	[21]
The elimination of errors due to baseline drift in the measurement of peak areas in gas chromatography	(Blank) Subtraction	1965	[20]
On a New Method of Graduation	Smoothing	1922	[27]
Background correction and multivariate curve resolution of online LC with IR detection.	MCR‐ALS	2011	[37]
Selectivity, local rank, three‐way data analysis and ambiguity in multivariate curve resolution	MCR‐ALS	1995	[34]
Mixture models for baseline estimation	Mixture model	2012	[49]
Morphology‐based automated baseline removal for Raman spectra of artistic pigments	Morphological correction	2010	[33]
Automatic correction of continuum background in Laser‐induced Breakdown Spectroscopy using a model‐free algorithm	Moving‐window minimum value	2014	[47]
Baseline correction using asymmetrically reweighted penalized least squares smoothing	Smoothing	2015	[32]
Baseline correction using adaptive iteratively reweighted penalized least squares	Smoothing	2010	[29]
A perfect smoother	Smoothing	2003	[24]
Morphological weighted penalized least squares for background correction	Smoothing	2013	[28]
Deconvolutions based on singular value decomposition and the pseudoinverse: a guide for beginners.	SVD	1994	[38]
Background removal from spectra by designing and minimising a non‐quadratic cost function	Backcor	2005	[53]
Image background removal in GC × GC	Image‐based correction	2003	[56]
A new approach to linear filtering and prediction problems	Smoothing	1960	[23]

Peak alignment			
Title	Subcategory	Year	Reference
Two‐dimensional correlation optimized warping algorithm for aligning GCxGC‐MS data	2D‐COW	2008	[66]
Retention time alignment for 2D data	2D alignment	2005	[76]
Automatic time‐shift alignment method for chromatographic data analysis	ATSA	2017	[69]
GC × GC retention time shift correction and modelling using bilinear peak alignment, correlation optimized shifting and MCR.	COSHIFT	2012	[63]
Alignment and clustering strategies for GC × GC‐MS features	Cylindrical mapping	2012	[80]
Retention time alignment to correct for wrap‐around	Wrap‐around	2005	[81]
Correlation optimized warping and dynamic time warping as preprocessing methods for chromatographic data	DTW	2004	[301]
Pixel‐by‐pixel correction of retention time shifts in chromatograms from GCxGC‐TOF‐MS	GC × GC	2017	[68]
Investigation of interpolation techniques for the reconstruction of the first dimension of LC × LC	Interpolation	2011	[79]
Automatic data analysis workflow for ultra‐high performance liquid chromatography‐high resolution MS‐based metabolomics	MS‐based alignment	2019	[72]
A chemometric‐assisted method based on gas chromatography‐mass spectrometry for metabolic profiling analysis	MS‐based alignment	2015	[73]
Robust algorithm for aligning two‐dimensional chromatograms	2D Alignment	2012	[67]
Handling within run retention time shifts in 2D data	PARAFRAC	2009	[74]
MS‐based peak alignment for automatic nontargeted metabolic profiling analysis for biomarker screening in plant samples	PCC	2017	[71]
Parametric Time Warping	PTW	2004	[62]
Peak alignment in LC × LC	LC × LC	2009	[57]

Peak detection			
Title	Subcategory	Year	Reference
Untargeted Peak detection in LC‐MS using Bayesian statistics	Bayesian statistics	2015	[119]
Probabilistic peak detection	Bayesian statistics	2014	[117]
Bayesian Approach for Peak Detection in Two‐Dimensional Chromatography	Bayesian statistics	2012	[115]
Comparative analysis of peak‐detection techniques for comprehensive two‐dimensional chromatography	Comparison	2011	[94]
Normal‐Gamma‐Bernoulli Peak Detection	Curve fitting	2017	[113]
Peak detection and background drift correction done with Curve fitting	Curve fitting	2014	[48]
Normal–exponential–Bernoulli based peak detection	Curve fitting	2014	[114]
Universal Denoising and Peak Picking Algorithm for LC−MS	Curve fitting	2003	[109]
Curve fitting using natural computation	Curve fitting	1994	[101]
Curve Fitting on overlapping peaks	Curve fitting	1994	[102]
Iterative curve fitting of chromatographic peaks	Curve fitting	1973	[103]
Characterization of exponentially modified Gaussian peaks in chromatography	Curve fitting	1972	[125]
Component selection for deconvolution	Deconvolution	2013	[105]
Peak detection and deconvolution of multi‐overlapped chromatographic signals	Derivative based	2005	[100]
LIMPIC: A computational method for the separation of protein MALDI‐TOF‐MS signals from noise	Moving‐average smoothing	2007	[45]
Improved peak detection and quantification of mass spectrometry data acquired from SELDI by denoising spectra with the undecimated discrete wavelet transform.	Wavelet transform	2005	[44]
Total Ion Spectra versus Segmented Total Ion Spectra as Preprocessing Tools for Gas Chromatography – Mass Spectrometry Data	Mass spectrometry	2018	[118]
Elastic Net Multivariate Curve Resolution Strategy for Sparse Spectral Recovery	MCR	2017	[142]
Peak detection coupled with multivariate curve resolution‐alternating least squares	MCR‐ALS	2019	[89]
Peak Detection and Deconvolution of Native Electrospray Mass Spectra from Large Protein Complexes	MS	2015	[108]
Peak clustering algorithm for comprehensive two‐dimensional liquid chromatography data analysis	Peak clustering	2019	[120]
Development of an algorithm for peak detection in comprehensive two‐dimensional chromatography	Peak clustering	2007	[4]
A comparison of three algorithms for chromatograms alignment	Peak clustering	2006	[134]
Peak Detection and Profiling from Multidimensional Chromatography	Review	2018	[98]
Peak detection methods for GC × GC	Review	2016	[97]
multi‐scale Gaussian smoothing‐based strategy for peak extraction	Smoothing	2016	[70]
Streak detection based on image analysis	Watershed	2018	[122]
Probability of failure of the watershed algorithm for peak detection in comprehensive two‐dimensional chromatography	Watershed	2010	[121]
Peak detection of TOF‐SIMS using continuous wavelet transform and curve fitting	Wavelet transform	2018	[302]
Recursive Wavelet Peak Detection	Wavelet transform	2016	[111]
Peak detection by continuous wavelet transform	Wavelet transform	2016	[107]
Multiscale peak detection in wavelet space	Wavelet transform	2015	[106]
Multiridge detection and time‐frequency reconstruction	Wavelet transform	1999	[110]

Peak properties			
Title	Subcategory	Year	Reference
Statistical moments in chromatography using trapezoidal and Simpson's rules of peak integration	Statistical moments	2019	[127]
Linearly modified Gaussian model	Curve models	2017	[126]
Comparison and optimization of different peak integration methods	Integration	2014	[129]
Asymmetric least squares baseline algorithm through the accuracy of statistical peak moments	Statistical moments	2013	[130]
Statistical moments in the exponentially modified Gaussian model of chromatography	Statistical moments	2003	[128]
Analysis of Peak Profiles Using Statistical Moments	Statistical moments	1995	[124]
Statistical theory of component overlap in multicomponent chromatograms	Curve fitting	1983	[116]
Computer characterization of chromatographic peaks by plate height and higher central moments	Statistical moments	1969	[123]

Data analysis			
Title	Subcategory	Year	Reference
Chromatographic response function for assessing the separation quality in comprehensive two‐dimensional liquid chromatography	CRF	2012	[99]
ANN in metabolomics	Deep learning	2019	[144]
Data analysis of metabolomic MS data	Mass spectrometry	2019	[89]
Multivariate Curve Resolution	MCR‐ALS	2014	[172]
Multivariate Data Analysis	Review	2014	[131]
Chemometrics for the analysis of chromatographic data in metabolomics investigations	Review	2014	[139]
Review of chemometric analysis techniques for comprehensive two‐dimensional separations data	Review	2012	[95]
Trends in data processing of comprehensive two‐dimensional chromatography	Review	2012	[93]
Recent advancements in comprehensive two‐dimensional separations with chemometrics	Review	2008	[280]
Multivariate Curve Resolution (MCR) from 2000: Progress in concepts and applications	Review	2006	[35]
De‐Tailing and Sharpening of Response peaks in Gas Chromatography	Peak Symmetrisation	1965	[83]
Power Law Approach as a Convenient Protocol for Improving Peak Shapes and Recovering Areas from Partially Resolved Peaks	Power Law	2019	[85]
Black box linearization for greater linear dynamic range: The effect of power transforms on the representation of data	Peak sharpening	2010	[84]
Comparison of wavelet transform and Fourier self‐deconvolution (FSD) and wavelet FSD for curve fitting	Fourier self‐deconvolution	2000	[86]
Filtering and deconvolution by the wavelet transform	Wavelet transform	1994	[87]

Data interpretation			
Title	Subcategory	Year	Reference
Peak evaluation by deep learning	Deep learning	2019	[145]
Multivariate data analysis	Multivariate data analysis	2014	[303]
Bin size effect on PCA	PCA	2020	[137]
Balancing Resolution with Analysis Time using PCA and the Mahalanobis Distance	PCA	2019	[136]
Discriminating Brazilian crude oils using comprehensive two‐dimensional gas chromatography‐mass spectrometry and multiway principal component analysis	PCA	2016	[304]
Differentiation of cocoa nibs	PCA	2016	[305]
Data evaluation in chromatography by principal component analysis	PCA	2010	[132]
Alignment of chromatographic profiles for principal component analysis	PCA	1994	[133]

Classification			
Title	Subcategory	Year	Reference
Application 0PLS‐DA authentication of fruit juice based on LC‐MS data	Application OPLS‐DA	2018	[154]
Application OPLS‐DA discrimination between pork meat and other meat sources based on LC‐MS data	Application OPLS‐DA	2018	[155]
Application PCA‐LDA chemometrics‐assisted HPLC‐DAD strategy for authentication of vintage year	Application PCA‐LDA	2017	[157]
Application PCA‐LDA for Geographical Origin Discrimination of Hazelnuts based on LC‐MS data	Application PCA‐LDA	2016	[156]
Application PLS‐DA Discrimination of genotype and geographical origin of black rice based on LC‐MS data	Application PLS‐DA	2019	[152]
Application PLS‐DA metabolomic study serum based biomarkers advanced melanoma based on LC‐MS and NMR data	Application PLS‐DA	2018	[151]
Application PLS‐DA GC × GC analysis of breath metabolome classification allergic asthma	Application PLS‐DA	2012	[153]
Application SIMCA detection of changes in N‐glycosylation profiles of therapeutic glycoproteins by LC‐MS	Application SIMCA	2017	[160]
Application SIMCA Geographical Origin Discrimination of edible palm oil by NP‐HPLC fingerprinting	Application SIMCA	2015	[159]
Application SVM's for classification based on GC‐MS metabolomics data	Application SVM's	2017	[163]
Application SVM's classification of edible vegetable oils based on GC‐MS data	Application SVM's	2016	[162]
Classification on mass chromatography by COW‐PCA‐LDA	COW‐PCA‐LDA	2018	[135]
DNN for classification of LC‐MS data	Deep learning	2019	[146]
Criteria for chemical identification with GC × GC‐MS	Peak selection	2005	[58]
Comparison of classification methods on GC × GC data	Method comparison	2019	[171]
Comparison of different classification methods, NIR data	Method comparison	2016	[306]
Comparison of classification methods on various data sets	Method comparison	2013	[147]
OPLS‐DA as an alternative to SIMCA and PLS‐DA	OPLS‐DA	2006	[307]
PLS‐DA, use, and methods	Review	2018	[148]
PLS‐DA, in metabolomics	Review	2015	[149]
PLS‐DA different methods and approaches	Review	2014	[308]
Unsupervised‐RF	RF	2016	[165]
Application RF combined with ACO, for the classification of the Greek olive oil varieties based on LC‐MS data	RF, ACO	2018	[166]
SIMCA description of method	SIMCA	2005	[148]
SVM's in chemometrics	SVM's	2006	[148]

Quantification			
Title	Subcategory	Year	Reference
Effect of background correction on peak detection and quantification in online comprehensive two‐dimensional liquid chromatography	AWLS	2012	[36]
Comparative study between univariate spectrophotometry and multivariate calibration	Comparison	2014	[309]
Comparison of multivariate curve resolution strategies in quantitative LC × LC	MCR	2017	[3]
Quantification with trilinear partial least squares for GC × GC	Tri‐PLS	2004	[75]
Chemometric approach to improve accuracy and precision of quantitation in liquid chromatography	MCR	2015	[141]
An initial estimation method using cosine similarity for MCR: Application to NMR spectra of chemical mixtures	MCR	2019	[39]
Determination of phenolic compounds by LC‐DAD	MCR‐ALS	2013	[177]
Chromatographic background drift correction coupled with parallel factor analysis to resolve coelution problems in 3D chromatographic data: Quantification of eleven antibiotics in tap water samples by high‐performance LC‐DAD	OSP	2013	[41]
Unique resolution of hidden minor peaks in multi‐detection chromatography by first‐order differentiation and orthogonal projections	OSP	1993	[40]
Resolution and quantification of peaks in liquid chromatography–mass spectrometry using PARAFAC2	PARAFAC2	2012	[140]
Targeted and non‐targeted sample profiling by GC × GC‐qMS	Profiling	2010	[78]
Peak detection and quantification of mass spectrometry data acquired from surface‐enhanced laser desorption and ionization by denoising spectra with the undecimated discrete wavelet transform	Wavelet transform	2005	[44]

Quantification co‐eluting compounds			
Title	Subcategory	Year	Reference
MCR tutorial	MCR‐ALS	2014	[172]
Deconvolution of overlapping spectral polymer signals in SEC by MCR‐ALS	MCR‐ALS	2014	[176]
Application MCR‐ALS coeluting compounds GC × GC analysis of Cannabis Sativa	MCR‐ALS	2014	[181]
Methods for initial guess in MCR‐ALS	MCR‐ALS, Comparison methods initial guess	1996	[174]
KSFA for initial guess in MCR‐ALS	MCR‐ALS, KSFA	1982	[175]
Comparison PARFAC and MCR methods on GC × GC	MCR‐ALS, PARFAC	2017	[180]
Comparison PARFAC and MCR methods on LC × LC	MCR‐ALS, PARFAC	2016	[179]
Simplisma for initial guess in MCR‐ALS	MCR‐ALS, SIMPLISMA	1991	[173]
Simultaneous deconvolution and re‐construction of primary and secondary overlapping peak clusters in GC × GC	NLLSCF	2011	[59]

Optimization			
Title	Subcategory	Year	Reference
Using computer modelling to predict and optimize separation in GC × GC	GC × GC	2008	[280]
Benefits of solvent concentration pulses in retention time modelling of LC	LC	2019	[282]
Enhancement in the computation of gradient retention times in LC using root‐finding methods	LC	2019	[285]
Gradient design for LC using multi‐scale optimizations	LC	2018	[284]
Method development using one‐segment‐per‐component optimization strategies	LC	2014	[238]
Optimization of HPLC with ANNs	LC	2005	[216]
Predictive kinetic optimization of HILIC × RP‐LC separations	LC × LC	2018	[11]
Program for the interpretive optimization of 2D resolution	LC × LC	2016	[286]
Optimization of conditions 2D‐RP‐LC	LC × LC	2015	[184]
Pareto‐optimality study into the comparison of LC × LC in the column and spatial mode	LC × LC	2012	[296]
Challenges in LC × LC	Review	2018	[5]
Optimizing separations in LC × LC	Review	2018	[6]
Optimization of GC × GC	Review	2012	[182]
Likelihood of total resolution in *s*LC × LC with parallel processing	*s*LC × LC	2018	[297]

Orthogonality			
Title	Subcategory	Year	Reference
Two metrics for measuring orthogonality for 2D chromatography	2D separations	2019	[263]
The role of surface coverage and orthogonality metrics for 2D chromatography	2D separations	2017	[249]
New method for the determination of peak distribution across a 2D separation space for optimal column combinations	2D separations	2016	[262]
Comparison of orthogonality metrics by statistical analysis	2D separations	2015	[257]
Comparison of orthogonality estimation methods for the 2D separation of peptides	2D separations	2015	[256]
Assessment of the orthogonality in 2D separative systems using criteria defined by the maximal information coefficient	2D separations	2015	[261]
Asterix equation: A new measure of orthogonality	2D separations	2014	[258]
Assessment of 2D separative systems using the nearest neighbour distances approach. Part 1: Orthogonality	2D separations	2013	[278]
A modelling approach for orthogonality of comprehensive 2D separations	2D separations	2013	[259]
Fractional coverage metrics based on ecological home range for calculating the effective peak capacity	2D separations	2012	[253]
The dimensionality of chromatographic separations	2D separations	2011	[252]
Orthogonality of 2D separations based on conditional entropy	2D separations	2011	[254]
Informational orthogonality of 2D chromatographic separations	2D separations	1996	[250]
Geometric approach to factor analysis for the estimation of orthogonality and practical peak capacity	2D separations	1995	[260]
Optimization of GC × GC method based on orthogonality	GC × GC	2018	[264]
Convex Hull: A new method to determine the used separation space	GC × GC	2010	[251]
Protocols for finding the most orthogonal dimensions for LC × LC	LC × LC	2015	[265]
Orthogonality measurement for multi‐dimensional chromatography in three and higher dimensional separations	Multi‐dimensional separations	2017	[266]

Resolution			
Title	Subcategory	Year	Reference
Valley‐to‐peak ratio as a measure for the separation of two chromatographic peaks	1D separations	1971	[267]
Development of a resolution metric for comprehensive 2D chromatography	2D separations	2007	[269]
Quantification of resolution for 2D separations	2D separations	1997	[268]

Peak capacity			
Title	Subcategory	Year	Reference
Chromatographic peak capacity and the factors influencing it	1D separations	1970	[271]
Maximum number of components resolvable in gel filtration and other elution chromatographic methods	1D separations	1967	[270]
Further considerations of exact equations for peak capacity in isocratic LC	LC	2014	[275]
Approximate and exact equations for peak capacity in isocratic HPLC	LC	2011	[274]
Study on the optimization of LC × LC considering losses in theoretical peak capacity in the ¹D and the ²D	LC × LC	2010	[187]

Chromatographic response functions			
Title	Subcategory	Year	Reference
Study on the performance of resolution criterion to characterize complex chromatograms	1D separations	2017	[277]
A chromatographic objective function to characterize chromatograms, peak prominence	1D separations	2015	[276]
Assessment of 2D separative systems using the nearest neighbour distances approach. Part 2: Separation quality aspect	2D separations	2013	[255]
Universal comparison of CRFs	LC	2014	[232]
A new CRF for assessing the separation quality in LC × LC	LC × LC	2012	[99]
CRFs in 1D and 2D chromatography as tools for assessing chemical complexity	Review	2013	[279]

Stationary phase selection			
Title	Subcategory	Year	Reference
Stationary phase selection in GC × GC	GC × GC	2012	[193]
Column selection with a multi‐column system and DryLab	LC	2006	[199]
Classification and comparison of RP‐LC columns using PCA	RP‐LC	2007	[195]
Characterization of RPLC columns with porous particles	RP‐LC	2007	[196]
Classification and comparison of RP‐LC columns	RP‐LC	2003	[194]

Retention modelling			
Title	Subcategory	Year	Reference
Prediction of retention time of capillary GC using MLR, PLS and back‐propagation ANNs	GC	2011	[207]
Influence of carrier gas on the prediction of GC retention times based on thermodynamic parameters	GC	2011	[220]
Prediction of GC retention time via an additive thermodynamic model	GC	2010	[219]
Evaluation of a structure‐driven retention model for temperature‐programmed GC	GC	2004	[218]
Prediction of GC retention indices using radial basis function ANNs	GC	2002	[208]
Regression algorithm for calculating ²D retention indices in GC × GC	GC × GC	2018	[222]
Retention time prediction in temperature‐programmed GC × GC	GC × GC	2014	[221]
QSRR‐based estimation of retention times in GC × GC	GC × GC	2012	[214]
Retention modelling in GC × GC using a QSRR model	GC × GC	2011	[211]
An accurate QSRR model for the prediction of GC × GC retention times	GC × GC	2007	[213]
Generating multiple independent retention index data in dual‐secondary GC × GC	GC × GC	2006	[224]
Estimation of environmental partitioning properties using GC × GC retention indices	GC × GC	2005	[223]
Prediction of protein retention times in gradient HIC	HIC	2008	[241]
Protein retention and selectivity in HIC using QSRR models	HIC	2006	[239]
New approaches for prediction of protein retention times in HIC	HIC	2006	[240]
Applicability of retention modelling in HILIC for algorithmic optimization programs	HILIC	2017	[234]
Retention modelling and method development in HILIC, PEWS	HILIC	2014	[231]
HILIC retention prediction under gradient elution	HILIC	2012	[237]
Study on the retention mechanism in HILIC ‐ Mixed mode model	HILIC	2011	[227]
Study on the retention equation in HILIC ‐ Mixed mode model	HILIC	2008	[228]
Development of an inorganic cations retention model in ion chromatography by means of ANNs	IEX	2005	[215]
Retention modelling in IEX ‐ Adsorption model	IEX	1996	[226]
Migration and elution equations in gradient LC	LC	2019	[287]
Simulation of elution profiles on columns with a stationary phase gradients	LC	2018	[206]
Simulation of elution profiles in LC: Gradient conditions, and with mismatching injection and mobile phase solvents	LC	2016	[288]
Data fitting problems encountered in modelling retention behaviour of analytes with dual retention mechanisms	LC	2015	[233]
Prediction of retention time in high‐resolution anti‐doping screening data using ANNs	LC	2013	[210]
Linear gradient prediction algorithm for stationary phase optimized selectivity LC	LC	2010	[204]
Simulation of elution profiles in LC: Investigation of the injection solvent in the second dimension	LC × LC	2017	[289]
Sorption of organic compounds on black carbon	LFER	2018	[246]
Application of hydrogen bonding calculations, LFER	LFER	2002	[244]
Effect of temperature on retention using RP‐LC	LFER, van ’t Hoff	2019	[247]
Optimization of ANNs for modelling impurities retention in micellar LC	MLC	2011	[209]
Retention modelling in NP‐LC and RP‐LC ‐ Adsorption model	NP‐LC, RP‐LC	2000	[225]
Applications of polyparameter LFER in environmental chemistry	Review, LFER	2014	[248]
Gradient retention time predictions for suspect screening	RP‐LC	2016	[217]
Improved RP gradient retention modelling, Neue‐Kuss model	RP‐LC	2010	[230]
Nonlinear retention relationships in RP‐LC, Neue‐Kuss model	RP‐LC	2006	[229]
Possibilities of retention modelling and computer‐assisted method development in SFC	SFC	2015	[238]

Peak tracking			
Title	Subcategory	Year	Reference
Bayesian peak tracking in GC × GC	GC × GC	2016	[295]
Peak‐tracking algorithm for use in automated interpretive method‐development tools in LC	LC	2018	[294]
Combined use of algorithms for peak picking, peak tracking, and retention modelling	LC	2011	[293]
Feature detection and alignment of LC‐MS data using Kalman tracking	LC	2008	[292]
Mutual peak alignment in series of HPLC‐DAD mixture analytes	LC	2003	[291]
Peak tracking of peptides in RP‐LC using DAD data	RP‐LC	1994	[290]

Method transfer			
Title	Subcategory	Year	Reference
Method transfer after changing pore diameter	LC	2019	[188]
Method transfer for fast LC ‐ Gradient experiments	LC	2008	[189]
Method transfer for fast LC ‐ Isocratic measurements	LC	2007	[190]

Feature selection			
Title	Subcategory	Year	Reference
Application ACO for feature selection of MALDI‐TOF data	ACO	2007	[168]
Ant colony optimization	ACO	2006	[310]
Application RF combined with ACO, for the classification of the Greek olive oil varieties based on LC‐MS data	RF, ACO	2018	[166]

Miscellaneous			
Title	Subcategory	Year	Reference
Bayesian regularization of neural networks	Bayesian statistics	2008	[50]
Application of GC × GC combined with pixel‐based chemometric processing for the chemical profiling of illicit drug samples	COW application	2008	[64]
Application of parallel computing to speed up chemometrics for GC × GC‐TOFMS based metabolic fingerprinting	Parallel computing	2011	[65]
Chemometrics‐assisted method development in RP‐LC	Review	2013	[185]
Recent developments in GC × GC	Review	2006	[192]
Tutorial on Bayesian chemometrics	Review	2007	[96]
The ‘PRISMA’ mobile phase optimization model in TLC	TLC	1985	[198]

## 6 CONCLUSIONS AND OUTLOOK

Robust data analysis strategies are needed to obtain useful information on complex samples using the increasingly advanced analytical tools. Preprocessing of the data is indispensable to remove irrelevant anomalies, which otherwise may induce significant errors in, for example, quantification or classification. For background correction, BEADS (baseline estimation and denoising using sparsity) and assisted BEADs are highly promising recent developments, as these approaches seems capable of handling many different types of background distortions and are fast. The main downside is that these are parametric methods that require prior optimization [63, 64]. An important development that may lead to more‐accurate information is the use of profile spectra instead of centroid spectra in the correction of GC–LC–MS data, which is especially important considering the prevalence of these hyphenated MS methods [55]. Along similar lines, the most noteworthy strategies for peak alignment in 2D chromatography are those that operate not just in one‐dimension but in both. Methods developed for such pixel‐level alignment are still quite scarce, especially for application in LC×LC. One such method has, however, been recently been developed for GC×GC–HRMS data by Zushi et al. [68].

Although there have been many additional interesting developments, it is difficult to judge which methods truly perform best. This is often better judged on a case‐by‐case basis. What has become abundantly clear is that a two‐dimensional chromatogram is still very often treated as a series of individual 1D chromatograms, with the preprocessing methods being applied separately to all of these. This is most likely because many of the existing methods have been developed for LC–MS data sets, rather than for two‐dimensional data. In terms of background correction, improvements can quite possibly be made by focusing on a series of modulations. The surface of the chromatogram may then be corrected, instead of applying a 1D method iteratively (row or column‐wise) to the data. There is a distinct lack of peak‐alignment strategies that can deal with changes in elution order.

Data analysis strategies, aimed to extract relevant information, are also rather difficult to compare, because the results greatly depend on the quality of the data. Most reported methods were developed to tackle a specific challenge in a data set and comparisons with other approaches supported by numerical data have rarely been reported. A comprehensive study of different types of data and data‐analysis techniques would allow a better overview of which techniques can be best used in which situation.

The water‐shed algorithm is often outperformed by the other techniques, but it may find new application in the field of polymer analysis. Polymer separations typically do not yield individually separated components (i.e. peaks), but envelopes or distributions (sometimes called “smears”), which are difficult to treat with curve‐fitting or derivative‐based methods.

Interest in deep‐learning methods is rapidly growing in other fields of science and algorithms are starting to be applied in chromatography, concurrently with the increase in computation power available. Deep learning methods are very flexible but limited to situations that are sufficiently represented in the training data.

Traditional classification methods such as PCA‐LDA, SIMCA and PLS‐DA are still most commonly applied, although newer methods, such as RF and SVM methods, are gaining popularity. Again, the different variations of each of these methods render an absolute comparison challenging. Numerical data are not yet available. However, RF and SVM methods have been shown to perform as well or even better than classical methods in some cases [147, 306]. Therefore, further studies in this direction are encouraged.

Quantification of compounds based on spectrally aided deconvolution is currently best performed using bilinear MCR‐ALS methods. Current multidimensional chromatographic methods fail to reconstruct 3D data with trilinear models. Future peak‐alignment models may be sufficiently accurate for this purpose.

Finally, almost all the discussed algorithms and tools for method optimization assume the optimum to concur with a maximum in one of the quality descriptors, such as peak capacity. The optimum, however, always depends on the objective of the optimization. The highest peak capacity, resolution or orthogonality may not always be required. In some cases, only a single compound is of interest and the only objective is to have this fully resolved from its neighboring peaks. In other cases, impurity profiling may be desired or obtaining a structured chromatogram may be the goal. For these reasons, the answer to the question “What is the true optimum of a separation?” is still debatable and incorporation of a user‐specified desirability parameter is recommended in future developments.

## CONFLICT OF INTEREST

The authors have declared no conflict of interest.
